# A systematic review of experimentally tested implementation strategies across health and human service settings: evidence from 2010-2022

**DOI:** 10.1186/s13012-024-01369-5

**Published:** 2024-06-24

**Authors:** Laura Ellen Ashcraft, David E. Goodrich, Joachim Hero, Angela Phares, Rachel L. Bachrach, Deirdre A. Quinn, Nabeel Qureshi, Natalie C. Ernecoff, Lisa G. Lederer, Leslie Page Scheunemann, Shari S. Rogal, Matthew J. Chinman

**Affiliations:** 1 Center for Health Equity Research and Promotion, Corporal Michael Crescenz VA Medical Center, Philadelphia, PA USA; 2 Department of Biostatistics, Epidemiology, and Informatics, University of Pennsylvania Perelman School of Medicine, Philadelphia, PA USA; 3 Center for Health Equity Research and Promotion, VA Pittsburgh Healthcare System, Pittsburgh, PA USA; 4 Division of General Internal Medicine, Department of Medicine, University of Pittsburgh, Pittsburgh, PA USA; 5 Clinical & Translational Science Institute, University of Pittsburgh, Pittsburgh, PA USA; 6 RAND Corporation, Pittsburgh, PA USA; 7 Center for Clinical Management Research, VA Ann Arbor Healthcare System, Ann Arbor, Michigan USA; 8 Department of Psychiatry, University of Michigan Medical School, Ann Arbor, MI USA; 9 Division of Geriatric Medicine, University of Pittsburgh, Department of Medicine, Pittsburgh, PA USA; 10 Division of Pulmonary, Allergy, Critical Care, and Sleep Medicine, University of Pittsburgh, Department of Medicine, Pittsburgh, PA USA; 11 Departments of Medicine and Surgery, University of Pittsburgh, Pittsburgh, Pennsylvania USA

**Keywords:** Implementation strategy, Systematic review, Health, Health-related outcomes

## Abstract

**Background:**

Studies of implementation strategies range in rigor, design, and evaluated outcomes, presenting interpretation challenges for practitioners and researchers. This systematic review aimed to describe the body of research evidence testing implementation strategies across diverse settings and domains, using the Expert Recommendations for Implementing Change (ERIC) taxonomy to classify strategies and the Reach Effectiveness Adoption Implementation and Maintenance (RE-AIM) framework to classify outcomes.

**Methods:**

We conducted a systematic review of studies examining implementation strategies from 2010-2022 and registered with PROSPERO (CRD42021235592). We searched databases using terms “implementation strategy”, “intervention”, “bundle”, “support”, and their variants. We also solicited study recommendations from implementation science experts and mined existing systematic reviews. We included studies that quantitatively assessed the impact of at least one implementation strategy to improve health or health care using an outcome that could be mapped to the five evaluation dimensions of RE-AIM. Only studies meeting prespecified methodologic standards were included. We described the characteristics of studies and frequency of implementation strategy use across study arms. We also examined common strategy pairings and cooccurrence with significant outcomes.

**Findings:**

Our search resulted in 16,605 studies; 129 met inclusion criteria. Studies tested an average of 6.73 strategies (0-20 range). The most assessed outcomes were Effectiveness (*n*=82; 64%) and Implementation (*n*=73; 56%). The implementation strategies most frequently occurring in the experimental arm were Distribute Educational Materials (*n*=99), Conduct Educational Meetings (*n*=96), Audit and Provide Feedback (*n*=76), and External Facilitation (*n*=59). These strategies were often used in combination. Nineteen implementation strategies were frequently tested and associated with significantly improved outcomes. However, many strategies were not tested sufficiently to draw conclusions.

**Conclusion:**

This review of 129 methodologically rigorous studies built upon prior implementation science data syntheses to identify implementation strategies that had been experimentally tested and summarized their impact on outcomes across diverse outcomes and clinical settings. We present recommendations for improving future similar efforts.

**Supplementary Information:**

The online version contains supplementary material available at 10.1186/s13012-024-01369-5.

Contributions to the literature

- While many implementation strategies exist, it has been challenging to compare their effectiveness across a wide range of trial designs and practice settings
- This systematic review provides a transdisciplinary evaluation of implementation strategies across population, practice setting, and evidence-based interventions using a standardized taxonomy of strategies and outcomes.
- Educational strategies were employed ubiquitously; nineteen other commonly used implementation strategies, including External Facilitation and Audit and Provide Feedback, were associated with positive outcomes in these experimental trials.
- This review offers guidance for scholars and practitioners alike in selecting implementation strategies and suggests a roadmap for future evidence generation.

## Background

Implementation strategies are “methods or techniques used to enhance the adoption, implementation, and sustainment of evidence-based practices or programs” (EBPs) [1]. In 2015, the Expert Recommendations for Implementing Change (ERIC) study organized a panel of implementation scientists to compile a standardized set of implementation strategy terms and definitions [2–4]. These 73 strategies were then organized into nine “clusters” [5]. The ERIC taxonomy has been widely adopted and further refined [6–13]. However, much of the evidence for individual or groups of ERIC strategies remains narrowly focused. Prior systematic reviews and meta-analyses have assessed strategy effectiveness, but have generally focused on a specific strategy, (e.g., Audit and Provide Feedback) [14–16], subpopulation, disease (e.g., individuals living with dementia) [16], outcome [15], service setting (e.g., primary care clinics) [17–19] or geography [20]. Given that these strategies are intended to have broad applicability, there remains a need to understand how well implementation strategies work across EBPs and settings and the extent to which implementation knowledge is generalizable.

There are challenges in assessing the evidence of implementation strategies across many EBPs, populations, and settings. Heterogeneity in population characteristics, study designs, methods, and outcomes have made it difficult to quantitatively compare which strategies work and under which conditions [21]. Moreover, there remains significant variability in how researchers operationalize, apply, and report strategies (individually or in combination) and outcomes [21, 22]. Still, synthesizing data related to using individual strategies would help researchers replicate findings and better understand possible mediating factors including the cost, timing, and delivery by specific types of health providers or key partners [23–25]. Such an evidence base would also aid practitioners with implementation planning such as when and how to deploy a strategy for optimal impact.

Building upon previous efforts, we therefore conducted a systematic review to evaluate the level of evidence supporting the ERIC implementation strategies across a broad array of health and human service settings and outcomes, as organized by the evaluation framework, RE-AIM (Reach, Effectiveness, Adoption, Implementation, Maintenance) [26–28]. A secondary aim of this work was to identify patterns in scientific reporting of strategy use that could not only inform reporting standards for strategies but also the methods employed in future. The current study was guided by the following research questions^1^:

1. What implementation strategies have been most commonly and rigorously tested in health and human service settings?
2. Which implementation strategies were commonly paired?
3. What is the evidence supporting commonly tested implementation strategies?

## Methods

We used the Preferred Reporting Items for Systematic Reviews and Meta-Analyses (PRISMA-P) model [29–31] to develop and report on the methods for this systematic review (Additional File 1). This study was considered to be non-human subjects research by the RAND institutional review board.

### Registration

The protocol was registered with PROSPERO (PROSPERO 2021 CRD42021235592).

### Eligibility criteria

This review sought to synthesize evidence for implementation strategies from research studies conducted across a wide range of health-related settings and populations. Inclusion criteria required studies to: 1) available in English; 2) published between January 1, 2010 and September 20, 2022; 3) based on experimental research (excluded protocols, commentaries, conference abstracts, or proposed frameworks); 4) set in a health or human service context (described below); 5) tested at least one quantitative outcome that could be mapped to the RE-AIM evaluation framework [26–28]; and 6) evaluated the impact of an implementation strategy that could be classified using the ERIC taxonomy [2, 32]. We defined health and human service setting broadly, including inpatient and outpatient healthcare settings, specialty clinics, mental health treatment centers, long-term care facilities, group homes, correctional facilities, child welfare or youth services, aging services, and schools, and required that the focus be on a health outcome. We excluded hybrid type I trials that primarily focused on establishing EBP effectiveness, qualitative studies, studies that described implementation barriers and facilitators without assessing implementation strategy impact on an outcome, and studies not meeting standardized rigor criteria defined below.

### Information sources

Our three-pronged search strategy included searching academic databases (i.e., CINAHL, PubMed, and Web of Science for replicability and transparency), seeking recommendations from expert implementation scientists, and assessing existing, relevant systematic reviews and meta-analyses.

### Search strategy

Search terms included “implementation strateg*” OR “implementation intervention*” OR “implementation bundl*” OR “implementation support*.” The search, conducted on September 20, 2022, was limited to English language and publication between 2010 and 2022, similar to other recent implementation science reviews [22]. This timeframe was selected to coincide with the advent of *Implementation Science* and when the term “implementation strategy” became conventionally used [2, 4, 33]. A full search strategy can be found in Additional File 2.

#### Title and abstract screening process

Each study’s title and abstract were read by two reviewers, who dichotomously scored studies on each of the six eligibility criteria described above as yes=1 or no=0, resulting in a score ranging from 1 to 6. Abstracts receiving a six from both reviewers were included in the full text review. Those with only one score of six were adjudicated by a senior member of the team (MJC, SSR, DEG). The study team held weekly meetings to troubleshoot and resolve any ongoing issues noted through the abstract screening process.

#### Full text screening

During the full text screening process, we reviewed, in pairs, each article that had progressed through abstract screening. Conflicts between reviewers were adjudicated by a senior member of the team for a final inclusion decision (MJC, SSR, DEG).

#### Review of study rigor

After reviewing published rigor screening tools [34–36], we developed an assessment of study rigor that was appropriate for the broad range of reviewed implementation studies. Reviewers evaluated studies on the following: 1) presence of a concurrent comparison or control group (=2 for traditional randomized controlled trial or stepped wedge cluster randomized trial and =1 for pseudo-randomized and other studies with concurrent control); 2) EBP standardization by protocol or manual (=1 if present); 3) EBP fidelity tracking (=1 if present); 4) implementation strategy standardization by operational description, standard training, or manual (=1 if present); 5) length of follow-up from full implementation of intervention (=2 for twelve months or longer, =1 for six to eleven months, or =0 for less than six months); and 6) number of sites (=1 for more than one site). Rigor scores ranged from 0 to 8, with 8 indicating the most rigorous. Articles were included if they 1) included a concurrent control group, 2) had an experimental design, and 3) received a score of 7 or 8 from two independent reviewers.

#### Outside expert consultation

We contacted 37 global implementation science experts who were recognized by our study team as leaders in the field or who were commonly represented among first or senior authors in the included abstracts. We asked each expert for recommendations of publications meeting study inclusion criteria (i.e., quantitatively evaluating the effectiveness of an implementation strategy). Recommendations were recorded and compared to the full abstract list.

#### Systematic reviews

Eighty-four systematic reviews were identified through the initial search strategy (See Additional File 3). Systematic reviews that examined the effectiveness of implementation strategies were reviewed in pairs for studies that were not found through our initial literature search.

### Data abstraction and coding

Data from the full text review were abstracted in pairs, with conflicts resolved by senior team members (DEG, MJC) using a standard Qualtrics abstraction form. The form captured the setting, number of sites and participants studied, evidence-based practice/program of focus, outcomes assessed (based on RE-AIM), strategies used in each study arm, whether the study took place in the U.S. or outside of the U.S., and the findings (i.e., was there significant improvement in the outcome(s)?). We coded implementation strategies used in the Control and Experimental Arms. We defined the Control Arm as receiving the lowest number of strategies (which could mean zero strategies or care as usual) and the Experimental Arm as the most intensive arm (i.e., receiving the highest number of strategies). When studies included multiple Experimental Arms, the Experimental Arm with the least intensive implementation strategy(ies) was classified as “Control” and the Experimental Arm with the most intensive implementation strategy(ies) was classified as the “Experimental” Arm.

Implementation strategies were classified using standard definitions (MJC, SSR, DEG), based on minor modifications to the ERIC taxonomy [2–4]. Modifications resulted in 70 named strategies and were made to decrease redundancy and improve clarity. These modifications were based on input from experts, cognitive interview data, and team consensus [37] (See Additional File 4). Outcomes were then coded into RE-AIM outcome domains following best practices as recommended by framework experts [26–28]. We coded the RE-AIM domain of Effectiveness as either an assessment of the effectiveness of the EBP or the implementation strategy. We did not assess implementation strategy fidelity or effects on health disparities as these are recently adopted reporting standards [27, 28] and not yet widely implemented in current publications. Further, we did not include implementation costs as an outcome because reporting guidelines have not been standardized [38, 39].

### Assessment and minimization of bias

Assessment and minimization of bias is an important component of high-quality systematic reviews. The Cochrane Collaboration guidance for conducting high-quality systematic reviews recommends including a specific assessment of bias for individual studies by assessing the domains of randomization, deviations of intended intervention, missing data, measurement of the outcome, and selection of the reported results (e.g., following a pre-specified analysis plan) [40, 41]. One way we addressed bias was by consolidating multiple publications from the same study into a single finding (i.e., *N*=1), so-as to avoid inflating estimates due to multiple publications on different aspects of a single trial. We also included high-quality studies only, as described above. However, it was not feasible to consistently apply an assessment of bias tool due to implementation science’s broad scope and the heterogeneity of study design, context, outcomes, and variable measurement, etc. For example, most implementation studies reviewed had many outcomes across the RE-AIM framework, with no one outcome designated as primary, precluding assignment of a single score across studies.

### Analysis

We used descriptive statistics to present the distribution of health or healthcare area, settings, outcomes, and the median number of included patients and sites per study, overall and by country (classified as U.S. vs. non-U.S.). Implementation strategies were described individually, using descriptive statistics to summarize the frequency of strategy use “overall” (in any study arm), and the mean number of strategies reported in the Control and Experimental Arms. We additionally described the strategies that were *only* in the experimental (and not control) arm, defining these as strategies that were “tested” and may have accounted for differences in outcomes between arms.

We described frequencies of pair-wise combinations of implementation strategies in the Experimental Arm. To assess the strength of the evidence supporting implementation strategies that were used in the Experimental Arm, study outcomes were categorized by RE-AIM and coded based on whether the association between use of the strategies resulted in a significantly positive effect (yes=1; no=0). We then created an indicator variable if at least one RE-AIM outcome in the study was significantly positive (yes=1; no=0). We plotted strategies on a graph with quadrants based on the combination of median number of studies in which a strategy appears and the median percent of studies in which a strategy was associated with at least one positive RE-AIM outcome. The upper right quadrant—higher number of studies overall and higher percent of studies with a significant RE-AIM outcome—represents a superior level of evidence. For implementation strategies in the upper right quadrant, we describe each RE-AIM outcome and the proportion of studies which have a significant outcome.

## Results

### Search results

**Fig. 1:**
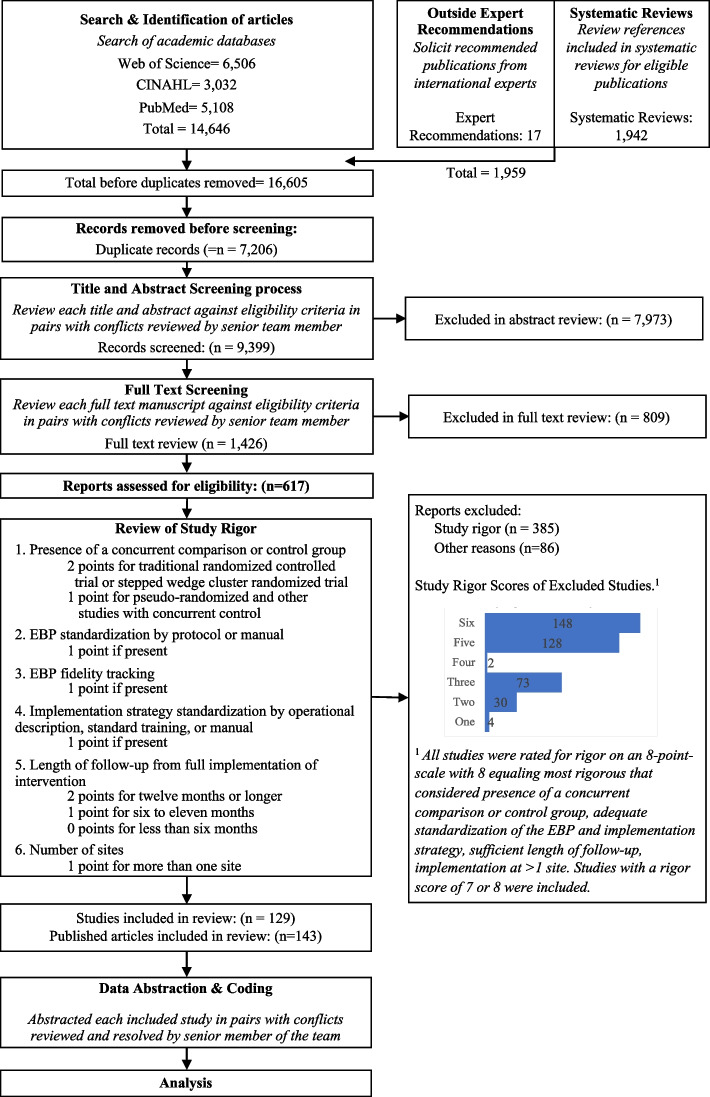
Expanded PRISMA Flow Diagram The expanded PRISMA flow diagram provides a description of each step in the review and abstraction process for the systematic review

We identified 14,646 articles through the initial literature search, 17 articles through expert recommendation (three of which were not included in the initial search), and 1,942 articles through reviewing prior systematic reviews (Fig. 1). After removing duplicates, 9,399 articles were included in the initial abstract screening. Of those, 48% (*n*=4,075) abstracts were reviewed in pairs for inclusion. Articles with a score of five or six were reviewed a second time (*n*=2,859). One quarter of abstracts that scored lower than five were reviewed for a second time at random. We screened the full text of 1,426 articles in pairs. Common reasons for exclusion were 1) study rigor, including no clear delineation between the EBP and implementation strategy, 2) not testing an implementation strategy, and 3) article type that did not meet inclusion criteria (e.g., commentary, protocol, etc.). Six hundred seventeen articles were reviewed for study rigor with 385 excluded for reasons related to study design and rigor, and 86 removed for other reasons (e.g., not a research article). Among the three additional expert-recommended articles, one met inclusion criteria and was added to the analysis. The final number of studies abstracted was 129 representing 143 publications.

### Descriptive results

Of 129 included studies (Table 1; see also Additional File 5 for Summary of Included Studies), 103 (79%) were conducted in a healthcare setting. EBP health care setting varied and included primary care (*n*=46; 36%), specialty care (*n*=27; 21%), mental health (*n*=11; 9%), and public health (*n*=30; 23%), with 64 studies (50%) occurring in an outpatient health care setting. Studies included a median of 29 sites and 1,419 target population (e.g., patients or students). The number of strategies varied widely across studies, with Control Arms averaging approximately two strategies (Range = 0-20, including studies with no strategy in the comparison group) and Experimental Arms averaging eight strategies (Range = 1-21). Non-US studies (*n*=73) included more sites and target population on average, with an overall median of 32 sites and 1,531 patients assessed in each study.

**Table 1:** Study characteristics

	Total (*n*=129)	United States (*n*=56)	Non-U.S. (*n*=73)
Country (n, %)			
United States (U.S.)	56 (43%)		
Other Country^a^	28 (22%)		
Australia	18 (14%)		
The Netherlands	14 (11%)		
United Kingdom	7 (5%)		
Canada^b^	6 (5%)		
Area of health or healthcare (n,%)^c^			
Primary care	46 (36%)	21 (36%)	25 (34%)
Public health	30 (23%)	12 (21%)	18 (25%)
Specialty care	27 (21%)	10 (18%)	17 (23%)
Mental health	11 (9%)	8 (14%)	3 (4%)
Other	18 (14%)	5 (9%)	13 (18%)
Setting (n, %)^c^			
Outpatient/clinic	64 (50%)	32 (57%)	32(44%)
Inpatient/hospital—floors	20 (15%)	4 (7%)	16 (22%)
School	13 (10%)	1 (2%)	12 (16%)
Community center	7 (5%)	5 (9%)	2 (3%)
Intensive Care Unit (ICU)	5 (5%)	1 (2%)	4 (5%)
Emergency/urgent care	3 (2%)	1 (2%)	2 (3%)
Home-based	1 (1%)	0 (0%)	1 (1%)
Other	29 (23%)	15 (58%)	14 (19%)
Number of sites, Median (IQR)	29 (12-49)	24 (12-47)	32 (12-55)
Number of participants, Median (IQR)	1,419 (306-5,957)	1,152 (239-4,040)	1,531 (365-7,279)
Implementation Strategies			
Control Arm [mean (SD) range]	1.64 (2.86) 0-20	1.39 (2.30) 0-9	1.82 (3.23) 0-20
Experimental Arm [mean (SD) range]	8.33 (4.71) 1-21	8.66 (4.49) 1-20	8.07 (4.88) 1-21
Tested [mean (SD) range]	6.73 (4.45) 0-20	7.30 (4.46) 1-20	6.29 (4.43) 0-20
Outcome Assessed^d^ (n, %)			
Reach	31 (24%)	12 (21%)	19 (26%)
Effectiveness	82 (64%)	31 (55%)	51 (70%)
Adoption	33 (26%)	13 (23%)	20 (27%)
Implementation	73 (56%)	29 (52%)	44 (60%)
Maintenance	40 (31%)	18 (32%)	22 (30%)
Positive outcome^e^^,f^ (n, %)			
Reach	18 (58%)	6 (50%)	12 (63%)
Effectiveness	44 (54%)	14 (45%)	30 (59%)
Adoption	24 (73%)	10 (77%)	14 (70%)
Implementation	54 (74%)	19 (66%)	35 (80%)
Maintenance	23 (58%)	9 (50%)	14 (64%)

^a^Other Countries included: Sweden (*n*=4), Germany (*n*=4), France (*n*=3), Spain (*n*=2), Zimbabwe, Uganda, Switzerland, Portugal, Norway, Nigeria, Kenya, Japan, Finland, Denmark, The Bahamas, New Zealand and three multi-country studies: Senegal and Mali; Australia and New Zealand; and England, Sweden, the Netherlands, and Republic of Ireland.

^b^One study was conducted in both the U.S. and Canada with 7 sites in Canada and 1 U.S. site. This study was coded as being conducted in Canada

^c^Studies could occur in more than one area of health/healthcare and setting. The denominator for reported percentages is the total number studies reviewed by column (Total *N* = 129; U.S. *N* = 56; Non-U.S. *N* = 73)).

^d^The denominator for reported percentages is the total number studies reviewed by column (Total *N* = 129; U.S. *N* = 56; Non-U.S. *N* = 73)); studies could assess more than one outcome

^e^The denominator for reported percentages is the total number studies reviewed by column (Total *N* = 129; U.S. *N* = 56; Non-U.S. *N* = 73));

^f^Studies may have tested several measures of a RE-AIM outcome. The designation of “positive outcome” indicates studies with at least one measure of the RE-AIM outcome that reached statistical significance when comparing the Experimental to Control Arm. For example, if a study used several measures of Reach, the outcomes would be considered a positive outcome if at least one of those measures was statistically significant.

Organized by RE-AIM, the most evaluated outcomes were Effectiveness (*n* = 82, 64%) and Implementation (*n* = 73, 56%); followed by Maintenance (*n*=40; 31%), Adoption (*n*=33; 26%), and Reach (*n*=31; 24%). Most studies (*n* = 98, 76%) reported at least one significantly positive outcome. Adoption and Implementation outcomes showed positive change in three-quarters of studies (*n*=78), while Reach (*n*=18; 58%), Effectiveness (*n*=44; 54%), and Maintenance (*n*=23; 58%) outcomes evidenced positive change in approximately half of studies.

The following describes the results for each research question.

1. What implementation strategies have been most commonly and rigorously tested in health and human service settings?

Table 2 shows the frequency of studies within which an implementation strategy was used in the Control Arm, Experimental Arm(s), and tested strategies (those used exclusively in the Experimental Arm) grouped by strategy type, as specified by previous ERIC reports [2, 6].

**Table 2:** Frequency of ERIC implementation strategy use

Implementation Strategy	Overall	Control Arm (Least intensive arm)	Experimental Arm (Most intensive arm)	Tested^b^
Use evaluative and iterative strategies				
Assess for readiness and identify barriers and facilitators	43	5	43	38
Audit and provide feedback	76	13	76	63
Conduct cyclical small tests of change	22	1	22	21
Conduct local need assessment	20	4	20	16
Develop a formal implementation blueprint	41	5	42	36
Develop and implement tools for quality monitoring	29	4	29	25
Obtain and use patients/consumers and family feedback	5	0	5	5
Purposefully reexamine the implementation	33	3	33	30
Stage implementation scale up	1	1	1	0
Assess and redesign workflows^a^	19	2	19	17
Provide interactive assistance				
Centralize technical assistance	16	8	13	8
Internal Facilitation^a^	10	1	9	9
External Facilitation^a^	59	5	59	54
Provide clinical supervision	4	0	4	4
Provide local technical assistance	9	2	9	7
Create an online learning community^a^	8	1	8	7
Adapt and tailor to context				
Promote adaptability	38	2	37	36
Tailor strategies	43	2	43	41
Use data experts	0	0	0	0
Use data warehousing techniques	2	0	2	2
Develop stakeholder interrelationships				
Build a coalition	4	1	4	3
Capture and share local knowledge	13	2	13	11
Conduct local consensus discussions	19	2	19	17
Develop academic partnerships	1	0	1	1
Identify and prepare champions	22	1	22	21
Identify early adopters	0	0	0	0
Inform local opinion leaders	11	1	11	10
Involve executive boards	6	0	6	6
Model and simulate change	2	0	2	2
Obtain formal commitments	12	4	12	8
Organize clinician implementation team meetings	43	6	42	37
Promote network weaving	1	0	1	1
Recruit, designate, and train for leadership	19	2	19	17
Use advisory boards and workgroups	11	1	10	10
Visit other sites	1	0	1	1
Engage community resources outside the practice*	4	0	4	4
Train and educate stakeholders				
Conduct educational meetings	97	29	96	68
Conduct educational outreach visits	20	2	19	18
Conduct ongoing training	34	5	34	29
Create a learning collaborative	15	4	15	11
Distribute educational materials	100	51	99	49
Make training dynamic	20	3	20	17
Provide ongoing consultation	19	3	19	16
Shadow other experts	1	0	1	1
Use train-the-trainer strategies	10	2	9	8
Support clinicians				
Create new clinical teams	1	0	1	1
Develop resource sharing agreements	0	0	0	0
Facilitate relay of clinical data to providers	10	2	10	8
Remind clinicians	22	5	22	17
Revise professional roles	2	0	2	2
Engage consumers				
Increase demand	6	1	6	5
Intervene with patients/consumers to enhance uptake and adherence	18	8	16	10
Involve patients/consumers and family members	8	1	8	7
Prepare patients/consumers to be active participants	20	5	20	15
Use mass media	2	2	2	0
Utilize financial strategies				
Access new funding	4	0	4	4
Alter incentive/allowance structures	9	4	8	5
Alter patient/consumer fees	0	0	0	0
Develop disincentives	0	0	0	0
Fund and contract for the clinical innovation	1	1	1	0
Make billing easier	0	0	0	0
Place innovation on fee for service lists/formularies	1	0	1	1
Use capitated payments	0	0	0	0
Use other payment schemes	1	0	1	1
Change infrastructure				
Change accreditation or membership requirements	2	0	2	2
Change liability laws	0	0	0	0
Change record systems	12	3	12	9
Change service sites	0	0	0	0
Mandate change	5	1	5	4
Start a dissemination organization	0	0	0	0

^a^Indicates implementation strategies new to ERIC

^b^The Tested column includes only those strategies used exclusively in the Experimental Arm. Tested strategies may not be the difference between Experimental Arm and Control Arm as strategies may occur in the Control Arm but not the Experimental Arm

The following strategies were determined to be duplicative or subsumed in other strategies: Develop and Organize Quality Monitoring Systems, Facilitation, Develop an Implementation Glossary, Use an Implementation Advisor, Develop Educational Materials, Work with Educational Institutions, Change Physical Structure and equipment, and Create or Change Credentialing and/or Licensure Standards. See Additional File 3 for full details.

Ten implementation strategies were not used in any studies reviewed, including: Use Data Experts, Change Liability Laws, Change Service Sites, Start a Dissemination Organization, Identify Early Adopters, Develop Resource Sharing Agreements, Alter Patient/Consumer Fees, Make Billing Easier, Use Capitated Payments, and Develop Disincentives

We acknowledge that the term stakeholder can be problematic in that it connotes the violent power differential for indigenous populations

### Control arm

In about half the studies (53%; *n*=69), the Control Arms were “active controls” that included at least one strategy, with an average of 1.64 (and up to 20) strategies reported in control arms. The two most common strategies used in Control Arms were: Distribute Educational Materials (*n*=52) and Conduct Educational Meetings (*n*=30).

### Experimental arm

**Fig. 2:**
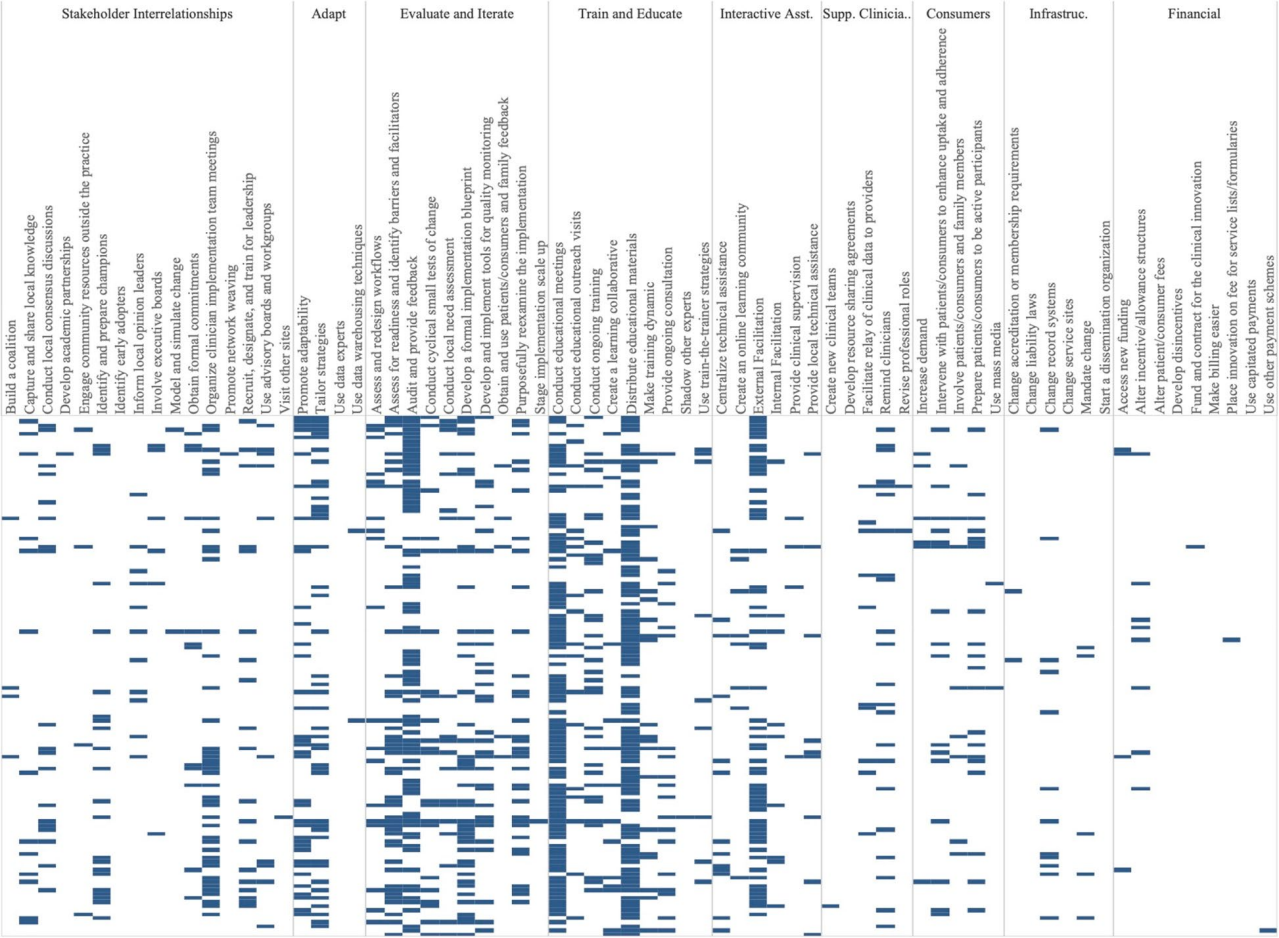
Implementation strategies used in the Experimental Arm of included studies. Explore more here: https://public.tableau.com/views/Figure2_16947070561090/Figure2?:language=en-US&:display_count=n&:origin=viz_share_link

Experimental conditions included an average of 8.33 implementation strategies per study (Range = 1-21). Figure 2 shows a heat map of the strategies that were used in the Experimental Arms in each study. The most common strategies in the Experimental Arm were Distribute Educational Materials (*n*=99), Conduct Educational Meetings (*n*=96), Audit and Provide Feedback (*n*=76), and External Facilitation (*n*=59).

### Tested strategies

The average number of implementation strategies that were included in the Experimental Arm only (and not in the Control Arm) was 6.73 (Range = 0-20).^2^ Overall, the top 10% of tested strategies included Conduct Educational Meetings (*n*=68), Audit and Provide Feedback (*n*=63), External Facilitation (*n*=54), Distribute Educational Materials (*n*=49), Tailor Strategies (*n*=41), Assess for Readiness and Identify Barriers and Facilitators (*n*=38) and Organize Clinician Implementation Team Meetings (*n*=37). Few studies tested a single strategy (*n*=9). These strategies included, Audit and Provide Feedback, Conduct Educational Meetings, Conduct Ongoing Training, Create a Learning Collaborative, External Facilitation (*n*=2), Facilitate Relay of Clinical Data To Providers, Prepare Patients/Consumers to be Active Participants, and Use Other Payment Schemes. Three implementation strategies were included in the Control or Experimental Arms but were not Tested including, Use Mass Media, Stage Implementation Scale Up, and Fund and Contract for the Clinical Innovation.

2. Which implementation strategies were commonly paired?

Table 3 shows the five most used strategies in Experimental Arms with their top ten most frequent pairings, excluding Distribute Educational Materials and Conduct Educational Meetings, as these strategies were included in almost all Experimental and half of Control Arms. The five most used strategies in the Experimental Arm included Audit and Provide Feedback (*n*=76), External Facilitation (*n*=59), Tailor Strategies (*n*=43), Assess for Readiness and Identify Barriers and Facilitators (*n*=43), and Organize Implementation Teams (*n*=42).

**Table 3:**
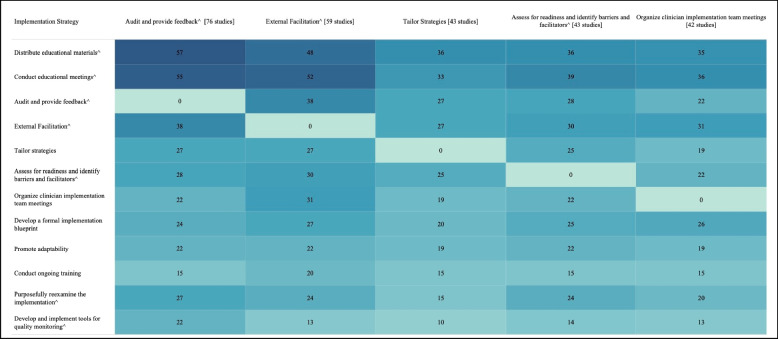
Top 5 commonly used strategies in the Experimental Arm and their 10 most common pairings, organized by cluster †

Darker colors indicate a high frequency of pairings between implementation strategies.

^**†**^These top 5 strategies exclude Distribute Educational Materials and Conduct Educational Meetings, as these strategies were included in almost all Experimental and half of Control arms.

**Fig. 3:**
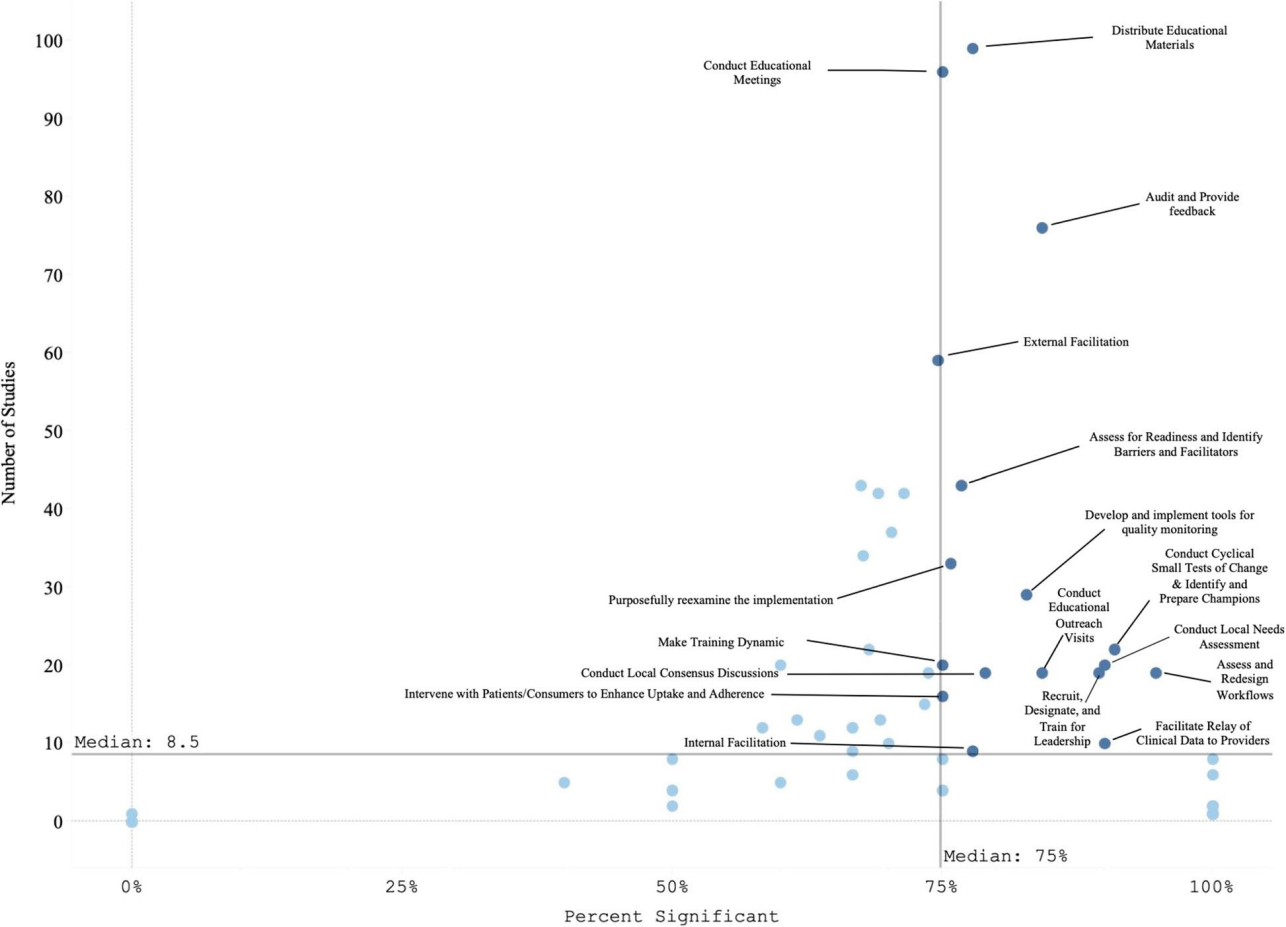
Experimental Arm Implementation Strategies with significant RE-AIM outcome. Explore more here: https://public.tableau.com/views/Figure3_16947017936500/Figure3?:language=en-US&publish=yes&:display_count=n&:origin=viz_share_link

^denotes an implementation strategy in the top right quadrant represented in Fig.3. Explore more here: https://public.tableau.com/views/Table3_16947882124200/Table3?:language=en-US&:display_count=n&:origin=viz_share_link

Strategies frequently paired with these five strategies included two educational strategies: Distribute Educational Materials and Conduct Educational Meetings. Other commonly paired strategies included Develop a Formal Implementation Blueprint, Promote Adaptability, Conduct Ongoing Training, Purposefully Reexamine the Implementation, and Develop and Implement Tools for Quality Monitoring.

3. What is the evidence supporting commonly tested implementation strategies?

We classified the strength of evidence for each strategy by evaluating both the number of studies in which each strategy appeared in the Experimental Arm and the percentage of times there was at least one significantly positive RE-AIM outcome. Using these factors, Fig. 3 shows the number of studies in which individual strategies were evaluated (on the y axis) compared to the percentage of times that studies including those strategies had at least one positive outcome (on the x axis). Due to the non-normal distribution of both factors, we used the median (rather than the mean) to create four quadrants. Strategies in the lower left quadrant were tested in fewer than the median number of studies (8.5) and were less frequently associated with a significant RE-AIM outcome (75%). The upper right quadrant included strategies that occurred in more than the median number of studies (8.5) and had more than the median percent of studies with a significant RE-AIM outcome (75%); thus those 19 strategies were viewed as having stronger evidence. Of those 19 implementation strategies, Conduct Educational Meetings, Distribute Educational Materials, External Facilitation, and Audit and Provide Feedback continued to occur frequently, appearing in 59-99 studies.

**Fig. 4:**
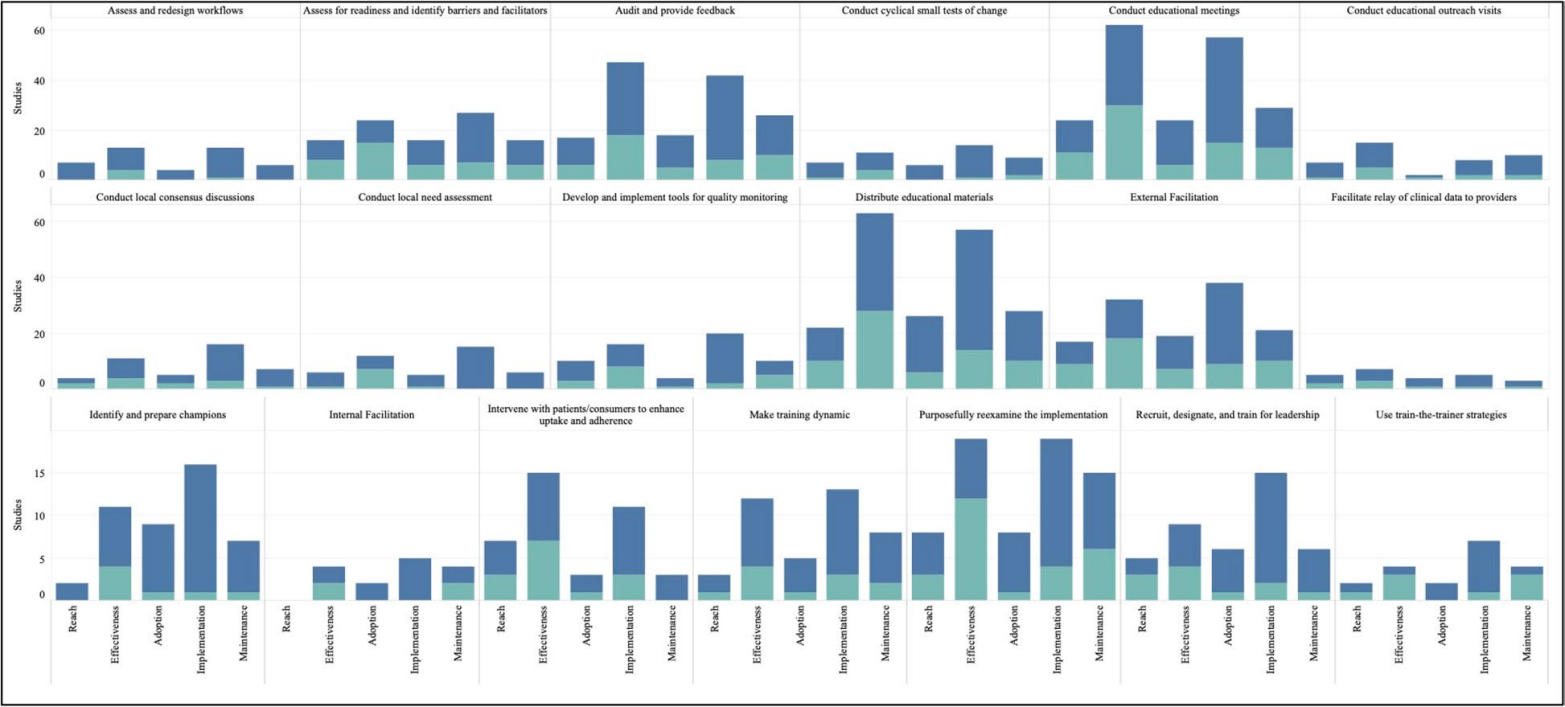
RE-AIM outcomes for the 19 Top-Right Quadrant Implementation Strategies**.** The y-axis is the number of studies and the x-axis is a stacked bar chart for each RE-AIM outcome with R=Reach, E=Effectiveness, A=Adoption, I=Implementation, M=Maintenance. Blue denotes at least one significant RE-AIM outcome; Light blue denotes studies which used the given implementation strategy and did not have a significant RE-AIM*.* Explore more here: https://public.tableau.com/views/Figure4_16947017112150/Figure4?:language=en-US&publish=yes&:display_count=n&:origin=viz_share_link

Figure 4 graphically illustrates the proportion of significant outcomes for each RE-AIM outcome for the 19 commonly used and evidence-based implementation strategies in the upper right quadrant. These findings again show the widespread use of Conduct Educational Meetings and Distribute Educational Materials. Implementation and Effectiveness outcomes were assessed most frequently, with Implementation being the mostly commonly reported significantly positive outcome.

## Discussion

This systematic review identified 129 experimental studies examining the effectiveness of implementation strategies across a broad range of health and human service studies. Overall, we found that evidence is lacking for most ERIC implementation strategies, that most studies employed combinations of strategies, and that implementation outcomes, categorized by RE-AIM dimensions, have not been universally defined or applied. Accordingly, other researchers have described the need for universal outcomes definitions and descriptions across implementation research studies [28, 42]. Our findings have important implications not only for the current state of the field but also for creating guidance to help investigators determine which strategies and in what context to examine.

The four most evaluated strategies were Distribute Educational Materials, Conduct Educational Meetings, External Facilitation, and Audit and Provide Feedback. Conducting Educational Meetings and Distributing Educational Materials were surprisingly the most common. This may reflect the fact that education strategies are generally considered to be “necessary but not sufficient” for successful implementation [43, 44]. Because education is often embedded in interventions, it is critical to define the boundary between the innovation and the implementation strategies used to support the innovation. Further specification as to when these strategies are EBP core components or implementation strategies (e.g., booster trainings or remediation) is needed [45, 46].

We identified 19 implementation strategies that were tested in at least 8 studies (more than the median) and were associated with positive results at least 75% of the time. These strategies can be further categorized as being used in early or pre-implementation versus later in implementation. Preparatory activities or pre-implementation, strategies that had strong evidence included educational activities (Meetings, Materials, Outreach visits, Train for Leadership, Use Train the Trainer Strategies) and site diagnostic activities (Assess for Readiness, Identify Barriers and Facilitators, Conduct Local Needs Assessment, Identify and Prepare Champions, and Assess and Redesign Workflows). Strategies that target the implementation phase include those that provide coaching and support (External and Internal Facilitation), involve additional key partners (Intervene with Patients to Enhance Uptake and Adherence), and engage in quality improvement activities (Audit and Provide Feedback, Facilitate the Relay of Clinical Data to Providers, Purposefully Reexamine the Implementation, Conduct Cyclical Small Tests of Change, Develop and Implement Tools for Quality Monitoring).

There were many ERIC strategies that were not represented in the reviewed studies, specifically the financial and policy strategies. Ten strategies were not used in any studies, including: Alter Patient/Consumer Fees, Change Liability Laws, Change Service Sites, Develop Disincentives, Develop Resource Sharing Agreements, Identify Early Adopters, Make Billing Easier, Start a Dissemination Organization, Use Capitated Payments, and Use Data Experts. One of the limitations of this investigation was that not all individual strategies or combinations were investigated. Reasons for the absence of these strategies in our review may include challenges with testing certain strategies experimentally (e.g., changing liability laws), limitations in our search terms, and the relative paucity of implementation strategy trials compared to clinical trials. Many “untested” strategies require large-scale structural changes with leadership support (see [47] for policy experiment example). Recent preliminary work has assessed the feasibility of applying policy strategies and described the challenges with doing so [48–50]. While not impossible in large systems like VA (for example: the randomized evaluation of the VA Stratification Tool for Opioid Risk Management) the large size, structure, and organizational imperative makes these initiatives challenging to experimentally evaluate. Likewise, the absence of these ten strategies may have been the result of our inclusion criteria, which required an experimental design. Thus, creative study designs may be needed to test high-level policy or financial strategies experimentally.

Some strategies that were likely under-represented in our search strategy included electronic medical record reminders and clinical decision support tools and systems. These are often considered “interventions” when used by clinical trialists and may not be indexed as studies involving ‘implementation strategies’ (these tools have been reviewed elsewhere [51–53]). Thus, strategies that are also considered interventions in the literature (e.g., education interventions) were not sought or captured. Our findings do not imply that these strategies are ineffective, rather that more study is needed. Consistent with prior investigations [54], few studies meeting inclusion criteria tested financial strategies. Accordingly, there are increasing calls to track and monitor the effects of financial strategies within implementation science to understand their effectiveness in practice [55, 56]. However, experts have noted that the study of financial strategies can be a challenge given that they are typically implemented at the system-level and necessitate research designs for studying policy-effects (e.g., quasi-experimental methods, systems-science modeling methods) [57]. Yet, there have been some recent efforts to use financial strategies to support EBPs that appear promising [58] and could be a model for the field moving forward.

The relationship between the number of strategies used and improved outcomes has been described inconsistently in the literature. While some studies have found improved outcomes with a bundle of strategies that were uniquely combined or a standardized package of strategies (e.g., Replicating Effective Programs [59, 60] and Getting To Outcomes [61, 62]), others have found that “more is not always better” [63–65]. For example, Rogal and colleagues documented that VA hospitals implementing a new evidence-based hepatitis C treatment chose >20 strategies, when multiple years of data linking strategies to outcomes showed that 1-3 specific strategies would have yielded the same outcome [39]. Considering that most studies employed multiple or multifaceted strategies, it seems that there is a benefit of using a targeted bundle of strategies that are purposefully aligns with site/clinic/population norms, rather than simply adding more strategies [66].

It is difficult to assess the effectiveness of any one implementation strategy in bundles where multiple strategies are used simultaneously. Even a ‘single’ strategy like External Facilitation is, in actuality, a bundle of narrowly constructed strategies (e.g., Conduct Educational Meetings, Identify and Prepare Champions, and Develop a Formal Implementation Blueprint). Thus, studying External Facilitation does not allow for a test of the individual strategies that comprise it, potentially masking the effectiveness of any individual strategy. While we cannot easily disaggregate the effects of multifaceted strategies, doing so may not yield meaningful results. Because strategies often synergize, disaggregated results could either underestimate the true impact of individual strategies or conversely, actually undermine their effectiveness (i.e., when their effectiveness comes from their combination with other strategies). The complexity of health and human service settings, imperative to improve public health outcomes, and engagement with community partners often requires the use of multiple strategies simultaneously. Therefore, the need to improve real-world implementation may outweigh the theoretical need to identify individual strategy effectiveness. In situations where it would be useful to isolate the impact of single strategies, we suggest that the same methods for documenting and analyzing the critical components (or core functions) of complex interventions [67–70] may help to identify core components of multifaceted implementation strategies [71–74].

In addition, to truly assess the impacts of strategies on outcomes, it may be necessary to track fidelity to implementation strategies (not just the EBPs they support). While this can be challenging, without some degree of tracking and fidelity checks, one cannot determine whether a strategy’s apparent failure to work was because it 1) was ineffective or 2) was not applied well. To facilitate this tracking there are pragmatic tools to support researchers. For example, the Longitudinal Implementation Strategy Tracking System (LISTS) offers a pragmatic and feasible means to assess fidelity to and adaptations of strategies [75].

## Implications for implementation science: four recommendations

Based on our findings, we offer four recommended “best practices” for implementation studies.

1. Prespecify strategies using standard nomenclature. This study reaffirmed the need to apply not only a standard *naming* convention (e.g., ERIC) but also a standard *reporting* of for implementation strategies. While reporting systems like those by Proctor [1] or Pinnock [75] would optimize learning across studies, few manuscripts specify strategies as recommended [76, 77]. Pre-specification allows planners and evaluators to assess the feasibility and acceptability of strategies with partners and community members [24, 78, 79] and allows evaluators and implementers to monitor and measure the fidelity, dose, and adaptations to strategies delivered over the course of implementation [27]. In turn, these data can be used to assess the costs, analyze their effectiveness [38, 80, 81], and ensure more accurate reporting [82–85]. This specification should include, among other data, the intensity, stage of implementation, and justification for the selection. Information regarding why strategies were selected for specific settings would further the field and be of great use to practitioners. [63, 65, 69, 79, 86].
2. Ensure that standards for measuring and reporting implementation outcomes are consistently applied and account for the complexity of implementation studies. Part of improving standardized reporting must include clearly defining outcomes and linking each outcome to particular implementation strategies. It was challenging in the present review to disentangle the impact of the intervention(s) (i.e., the EBP) versus the impact of the implementation strategy(ies) for each RE-AIM dimension. For example, often fidelity to the EBP was reported but not for the implementation strategies. Similarly, Reach and Adoption of the intervention would be reported for the Experimental Arm but not for the Control Arm, prohibiting statistical comparisons of strategies on the relative impact of the EBP between study arms. Moreover, there were many studies evaluating numerous outcomes, risking data dredging. Further, the significant heterogeneity in the ways in which implementation outcomes are operationalized and reported is a substantial barrier to conducting large-scale meta-analytic approaches to synthesizing evidence for implementation strategies [67]. The field could look to others in the social and health sciences for examples in how to test, validate, and promote a common set of outcome measures to aid in bringing consistency across studies and real-world practice (e.g., the NIH-funded Patient-Reported Outcomes Measurement Information System [PROMIS], https://www.healthmeasures.net/explore-measurement-systems/promis).
3. Develop infrastructure to learn cross-study lessons in implementation science. Data repositories, like those developed by NCI for rare diseases, U.S. HIV Implementation Science Coordination Initiative [87], and the Behavior Change Technique Ontology [88], could allow implementation scientists to report their findings in a more standardized manner, which would promote ease of communication and contextualization of findings across studies. For example, the HIV Implementation Science Coordination Initiative requested all implementation projects use common frameworks, developed user friendly databases to enable practitioners to match strategies to determinants, and developed a dashboard of studies that assessed implementation determinants [89–94].
4. Develop and apply methods to rigorously study common strategies and bundles. These findings support prior recommendations for improved empirical rigor in implementation studies [46, 95]. Many studies were excluded from our review based on not meeting methodological rigor standards. Understanding the effectiveness of discrete strategies deployed alone or in combination requires reliable and low burden tracking methods to collect information about strategy use and outcomes. For example, frameworks like the Implementation Replication Framework [96] could help interpret findings across studies using the same strategy bundle. Other tracking approaches may leverage technology (e.g., cell phones, tablets, EMR templates) [78, 97] or find novel, pragmatic approaches to collect recommended strategy specifications over time (e.g.., dose, deliverer, and mechanism) [1, 9, 27, 98, 99]. Rigorous reporting standards could inform more robust analyses and conclusions (e.g., moving toward the goal of understanding causality, microcosting efforts) [24, 38, 100, 101]. Such detailed tracking is also required to understand how site-level factors moderate implementation strategy effects [102]. In some cases, adaptive trial designs like sequential multiple assignment randomized trials (SMARTs) and just-in-time adaptive interventions (JITAIs) can be helpful for planning strategy escalation.

## Limitations

Despite the strengths of this review, there were certain notable limitations. For one, we only included experimental studies, omitting many informative observational investigations that cover the range of implementation strategies. Second, our study period was centered on the creation of the journal *Implementation Science* and not on the standardization and operationalization of implementation strategies in the publication of the ERIC taxonomy (which came later). This, in conjunction with latency in reporting study results and funding cycles, means that the employed taxonomy was not applied in earlier studies. To address this limitation, we retroactively mapped strategies to ERIC, but it is possible that some studies were missed. Additionally, indexing approaches used by academic databases may have missed relevant studies. We addressed this particular concern by reviewing other systematic reviews of implementation strategies and soliciting recommendations from global implementation science experts.

Another potential limitation comes from the ERIC taxonomy itself—i.e., strategy listings like ERIC are only useful when they are widely adopted and used in conjunction with guidelines for specifying and reporting strategies [1] in protocol and outcome papers. Although the ERIC paper has been widely cited (over three thousand times, accessed about 186 thousand times), it is still not universally applied, making tracking the impact of specific strategies more difficult. However, our experience with this review seemed to suggest that ERIC’s use was increasing over time. Also, some have commented that ERIC strategies can be unclear and are missing key domains. Thus, researchers are making definitions clearer for lay users [37, 103], increasing the number of discrete strategies for specific domains like HIV treatment, acknowledging strategies for new functions (e.g., de-implementation [104], local capacity building), accounting for phases of implementation (dissemination, sustainment [13], scale-up), addressing settings [12, 20], actors roles in the process, and making mechanisms of change to select strategies more user-friendly through searchable databases [9, 10, 54, 73, 104–106]. In sum, we found the utility of the ERIC taxonomy to outweigh any of the taxonomy’s current limitations.

As with all reviews, the search terms influenced our findings. As such, the broad terms for implementation strategies (e.g., “evidence-based interventions”[7] or “behavior change techniques” [107]) may have led to inadvertent omissions of studies of specific strategies. For example, the search terms may not have captured tests of policies, financial strategies, community health promotion initiatives, or electronic medical record reminders, due to differences in terminology used in corresponding subfields of research (e.g., health economics, business, health information technology, and health policy). To manage this, we asked experts to inform us about any studies that they would include and cross-checked their lists with what was identified through our search terms, which yielded very few additional studies. We included standard coding using the ERIC taxonomy, which was a strength, but future work should consider including the additional strategies that have been recommended to augment ERIC, around sustainment [13, 79, 106, 108], community and public health research [12, 109–111], consumer or service user engagement [112], de-implementation [104, 113–117] and related terms [118].

We were unable to assess the bias of studies due to non-standard reporting across the papers and the heterogeneity of study designs, measurement of implementation strategies and outcomes, and analytic approaches. This could have resulted in over- or underestimating the results of our synthesis. We addressed this limitation by being cautious in our reporting of findings, specifically in identifying “effective” implementation strategies. Further, we were not able to gather primary data to evaluate effect sizes across studies in order to systematically evaluate bias, which would be fruitful for future study.

## Conclusions

This novel review of 129 studies summarized the body of evidence supporting the use of ERIC-defined implementation strategies to improve health or healthcare. We identified commonly occurring implementation strategies, frequently used bundles, and the strategies with the highest degree of supportive evidence, while simultaneously identifying gaps in the literature. Additionally, we identified several key areas for future growth and operationalization across the field of implementation science with the goal of improved reporting and assessment of implementation strategies and related outcomes.

### Supplementary Information

Supplementary Material 1.

Supplementary Material 2.

Supplementary Material 3.

Supplementary Material 4.

Supplementary Material 5.

## Data Availability

All data for this study are included in this published article and its supplementary information files.

## Abbreviations

CDC: Centers for Disease Control
CINAHL: Cumulated Index to Nursing and Allied Health Literature
D&I: Dissemination and Implementation
EBP: Evidence-based practices or programs
ERIC: Expert Recommendations for Implementing Change
MOST: Multiphase Optimization Strategy
NCI: National Cancer Institute
NIH: National Institutes of Health
Pitt DISC: The Pittsburgh Dissemination and Implementation Science Collaborative
SMART: Sequential Multiple Assignment Randomized Trial
U.S.: United States
VA: Department of Veterans Affairs

## Footnotes

1 We modestly revised the following research questions from our PROSPERO registration after reading the articles and better understanding the nature of the literature: 1) What is the available evidence regarding the effectiveness of implementation strategies in supporting the uptake and sustainment of evidence intended to improve health and healthcare outcomes? 2) What are the current gaps in the literature (i.e., implementation strategies that do not have sufficient evidence of effectiveness) that require further exploration?

2 Tested strategies are those which exist in the Experimental Arm but not in the Control Arm. Comparative effectiveness or time staggered trials may not have any unique strategies in the Experimental Arm and therefore in our analysis would have no Tested Strategies.

## References

[1] Proctor EK, Powell BJ, McMillen JC. Implementation strategies: recommendations for specifying and reporting. Implement Sci. 2013;8:139. 24289295 10.1186/1748-5908-8-139 PMC3882890

[2] Powell BJ, Waltz TJ, Chinman MJ, Damschroder LJ, Smith JL, Matthieu MM, et al. A refined compilation of implementation strategies: results from the Expert Recommendations for Implementing Change (ERIC) project. Implement Sci. 2015;10:21. 25889199 10.1186/s13012-015-0209-1 PMC4328074

[3] Waltz TJ, Powell BJ, Chinman MJ, Smith JL, Matthieu MM, Proctor EK, et al. Expert recommendations for implementing change (ERIC): protocol for a mixed methods study. Implement Sci IS. 2014;9:39. 24669765 10.1186/1748-5908-9-39 PMC3987065

[4] Powell BJ, McMillen JC, Proctor EK, Carpenter CR, Griffey RT, Bunger AC, et al. A Compilation of Strategies for Implementing Clinical Innovations in Health and Mental Health. Med Care Res Rev. 2012;69:123–57. 22203646 10.1177/1077558711430690 PMC3524416

[5] Waltz TJ, Powell BJ, Matthieu MM, Damschroder LJ, Chinman MJ, Smith JL, et al. Use of concept mapping to characterize relationships among implementation strategies and assess their feasibility and importance: results from the Expert Recommendations for Implementing Change (ERIC) study. Implement Sci. 2015;10:109. 26249843 10.1186/s13012-015-0295-0 PMC4527340

[6] Perry CK, Damschroder LJ, Hemler JR, Woodson TT, Ono SS, Cohen DJ. Specifying and comparing implementation strategies across seven large implementation interventions: a practical application of theory. Implement Sci. 2019;14(1):32. 10.1186/s13012-019-0876-4 PMC6429753 30898133

[7] Community Preventive Services Task Force. Community Preventive Services Task Force: All Active Findings June 2023 [Internet]. 2023 [cited 2023 Aug 7]. Available from: https://www.thecommunityguide.org/media/pdf/CPSTF-All-Findings-508.pdf

[8] Solberg LI, Kuzel A, Parchman ML, Shelley DR, Dickinson WP, Walunas TL, et al. A Taxonomy for External Support for Practice Transformation. J Am Board Fam Med JABFM. 2021;34:32–9. 33452080 10.3122/jabfm.2021.01.200225 PMC9190131

[9] Leeman J, Birken SA, Powell BJ, Rohweder C, Shea CM. Beyond “implementation strategies”: classifying the full range of strategies used in implementation science and practice. Implement Sci. 2017;12:1–9. 29100551 10.1186/s13012-017-0657-x PMC5670723

[10] Leeman J, Calancie L, Hartman MA, Escoffery CT, Herrmann AK, Tague LE, et al. What strategies are used to build practitioners’ capacity to implement community-based interventions and are they effective?: a systematic review. Implement Sci. 2015;10:1–15. 26018220 10.1186/s13012-015-0272-7 PMC4449971

[11] Nathan N, Shelton RC, Laur CV, Hailemariam M, Hall A. Editorial: Sustaining the implementation of evidence-based interventions in clinical and community settings. Front Health Serv. 2023;3:1176023. 37033900 10.3389/frhs.2023.1176023 PMC10080155

[12] Balis LE, Houghtaling B, Harden SM. Using implementation strategies in community settings: an introduction to the Expert Recommendations for Implementing Change (ERIC) compilation and future directions. Transl Behav Med. 2022;12:965–78. 36039843 10.1093/tbm/ibac061

[13] Nathan N, Powell BJ, Shelton RC, Laur CV, Wolfenden L, Hailemariam M, et al. Do the Expert Recommendations for Implementing Change (ERIC) strategies adequately address sustainment? Front Health Serv. 2022;2:905909. 36925827 10.3389/frhs.2022.905909 PMC10012683

[14] Ivers N, Jamtvedt G, Flottorp S, Young JM, Odgaard-Jensen J, French SD, et al. Audit and feedback effects on professional practice and healthcare outcomes. Cochrane Database Syst Rev. 2012;6:CD000259. 10.1002/14651858.CD000259.pub3 PMC11338587 22696318

[15] Moore L, Guertin JR, Tardif P-A, Ivers NM, Hoch J, Conombo B, et al. Economic evaluations of audit and feedback interventions: a systematic review. BMJ Qual Saf. 2022;31:754–67. 35750494 10.1136/bmjqs-2022-014727

[16] Sykes MJ, McAnuff J, Kolehmainen N. When is audit and feedback effective in dementia care? A systematic review. Int J Nurs Stud. 2018;79:27–35. 29128686 10.1016/j.ijnurstu.2017.10.013

[17] Barnes C, McCrabb S, Stacey F, Nathan N, Yoong SL, Grady A, et al. Improving implementation of school-based healthy eating and physical activity policies, practices, and programs: a systematic review. Transl Behav Med. 2021;11:1365–410. 34080618 10.1093/tbm/ibab037 PMC8320878

[18] Tomasone JR, Kauffeldt KD, Chaudhary R, Brouwers MC. Effectiveness of guideline dissemination and implementation strategies on health care professionals’ behaviour and patient outcomes in the cancer care context: a systematic review. Implement Sci. 2020;15:1–18. 32493348 10.1186/s13012-020-0971-6 PMC7268663

[19] Seda V, Moles RJ, Carter SR, Schneider CR. Assessing the comparative effectiveness of implementation strategies for professional services to community pharmacy: A systematic review. Res Soc Adm Pharm. 2022;18:3469–83. 10.1016/j.sapharm.2022.03.019 35688687

[20] Lovero KL, Kemp CG, Wagenaar BH, Giusto A, Greene MC, Powell BJ, et al. Application of the Expert Recommendations for Implementing Change (ERIC) compilation of strategies to health intervention implementation in low- and middle-income countries: a systematic review. Implement Sci. 2023;18:56. 37904218 10.1186/s13012-023-01310-2 PMC10617067

[21] Chapman A, Rankin NM, Jongebloed H, Yoong SL, White V, Livingston PM, et al. Overcoming challenges in conducting systematic reviews in implementation science: a methods commentary. Syst Rev. 2023;12:1–6. 37420258 10.1186/s13643-023-02285-3 PMC10327144

[22] Proctor EK, Bunger AC, Lengnick-Hall R, Gerke DR, Martin JK, Phillips RJ, et al. Ten years of implementation outcomes research: a scoping review. Implement Sci. 2023;18:1–19. 37491242 10.1186/s13012-023-01313-z PMC10367273

[23] Michaud TL, Pereira E, Porter G, Golden C, Hill J, Kim J, et al. Scoping review of costs of implementation strategies in community, public health and healthcare settings. BMJ Open. 2022;12:e060785. 35768106 10.1136/bmjopen-2022-060785 PMC9240875

[24] Sohn H, Tucker A, Ferguson O, Gomes I, Dowdy D. Costing the implementation of public health interventions in resource-limited settings: a conceptual framework. Implement Sci. 2020;15:1–8. 32993713 10.1186/s13012-020-01047-2 PMC7526415

[25] Peek C, Glasgow RE, Stange KC, Klesges LM, Purcell EP, Kessler RS. The 5 R’s: an emerging bold standard for conducting relevant research in a changing world. Ann Fam Med. 2014;12:447–55. 25354409 10.1370/afm.1688 PMC4157982

[26] Glasgow RE, Vogt TM, Boles SM. Evaluating the public health impact of health promotion interventions: the RE-AIM framework. Am J Public Health. 1999;89:1322–7. 10474547 10.2105/AJPH.89.9.1322 PMC1508772

[27] Shelton RC, Chambers DA, Glasgow RE. An Extension of RE-AIM to Enhance Sustainability: Addressing Dynamic Context and Promoting Health Equity Over Time. Front Public Health. 2020;8:134. 32478025 10.3389/fpubh.2020.00134 PMC7235159

[28] Holtrop JS, Estabrooks PA, Gaglio B, Harden SM, Kessler RS, King DK, et al. Understanding and applying the RE-AIM framework: Clarifications and resources. J Clin Transl Sci. 2021;5:e126. 34367671 10.1017/cts.2021.789 PMC8327549

[29] Moher D, Shamseer L, Clarke M, Ghersi D, Liberati A, Petticrew M, et al. Preferred reporting items for systematic review and meta-analysis protocols (PRISMA-P) 2015 statement. Syst Rev. 2015;4:1. 25554246 10.1186/2046-4053-4-1 PMC4320440

[30] Shamseer L, Moher D, Clarke M, Ghersi D, Liberati A, Petticrew M, et al. Preferred reporting items for systematic review and meta-analysis protocols (PRISMA-P) 2015: elaboration and explanation. BMJ. 2015;349:g7647. 10.1136/bmj.g7647 25555855

[31] Page MJ, McKenzie JE, Bossuyt PM, Boutron I, Hoffmann TC, Mulrow CD, et al. The PRISMA 2020 statement: an updated guideline for reporting systematic reviews. BMJ [Internet]. 2021;372. Available from: https://www.bmj.com/content/372/bmj.n71 10.1136/bmj.n71 PMC8005924 33782057

[32] Rabin BA, Brownson RC, Haire-Joshu D, Kreuter MW, Weaver NL. A Glossary for Dissemination and Implementation Research in Health. J Public Health Manag Pract. 2008;14:117–23. 18287916 10.1097/01.PHH.0000311888.06252.bb

[33] Eccles MP, Mittman BS. Welcome to Implementation Science. Implement Sci. 2006;1:1. 10.1186/1748-5908-1-1

[34] Miller WR, Wilbourne PL. Mesa Grande: a methodological analysis of clinical trials of treatments for alcohol use disorders. Addict Abingdon Engl. 2002;97:265–77. 10.1046/j.1360-0443.2002.00019.x 11964100

[35] Miller WR, Brown JM, Simpson TL, Handmaker NS, Bien TH, Luckie LF, et al. What works? A methodological analysis of the alcohol treatment outcome literature. Handb Alcohol Treat Approaches Eff Altern 2nd Ed. Needham Heights, MA, US: Allyn & Bacon; 1995:12–44.

[36] Wells S, Tamir O, Gray J, Naidoo D, Bekhit M, Goldmann D. Are quality improvement collaboratives effective? A systematic review BMJ Qual Saf. 2018;27:226–40. 29055899 10.1136/bmjqs-2017-006926

[37] Yakovchenko V, Chinman MJ, Lamorte C, Powell BJ, Waltz TJ, Merante M, et al. Refining Expert Recommendations for Implementing Change (ERIC) strategy surveys using cognitive interviews with frontline providers. Implement Sci Commun. 2023;4:1–14. 37085937 10.1186/s43058-023-00409-3 PMC10122282

[38] Wagner TH, Yoon J, Jacobs JC, So A, Kilbourne AM, Yu W, et al. Estimating costs of an implementation intervention. Med Decis Making. 2020;40:959–67. 33078681 10.1177/0272989X20960455

[39] Gold HT, McDermott C, Hoomans T, Wagner TH. Cost data in implementation science: categories and approaches to costing. Implement Sci. 2022;17:11. 35090508 10.1186/s13012-021-01172-6 PMC8796347

[40] Boutron I, Page MJ, Higgins JP, Altman DG, Lundh A, Hróbjartsson A. Considering bias and conflicts of interest among the included studies. In: Higgins JPT, Thomas J, Chandler J, Cumpston M, Li T, Page MJ, Welch VA, editors. Cochrane Handbook for Systematic Reviews of Interventions. 2019. 10.1002/9781119536604.ch7.

[41] Higgins JP, Savović J, Page MJ, Elbers RG, Sterne J. Assessing risk of bias in a randomized trial. Cochrane Handb Syst Rev Interv. 2019;6:205–28. 10.1002/9781119536604.ch8

[42] Reilly KL, Kennedy S, Porter G, Estabrooks P. Comparing, Contrasting, and Integrating Dissemination and Implementation Outcomes Included in the RE-AIM and Implementation Outcomes Frameworks. Front Public Health [Internet]. 2020 [cited 2024 Apr 24];8. Available from: https://www.frontiersin.org/journals/public-health/articles/10.3389/fpubh.2020.00430/full 10.3389/fpubh.2020.00430 PMC7492593 32984239

[43] Grimshaw JM, Thomas RE, MacLennan G, Fraser C, Ramsay CR, Vale L, et al. Effectiveness and efficiency of guideline dissemination and implementation strategies. Health Technol Assess Winch Engl. 2004;8:iii–iv 1-72. 10.3310/hta8060 14960256

[44] Beidas RS, Kendall PC. Training Therapists in Evidence-Based Practice: A Critical Review of Studies From a Systems-Contextual Perspective. Clin Psychol Publ Div Clin Psychol Am Psychol Assoc. 2010;17:1–30. 10.1111/j.1468-2850.2009.01187.x PMC2945375 20877441

[45] Powell BJ, Beidas RS, Lewis CC, Aarons GA, McMillen JC, Proctor EK, et al. Methods to Improve the Selection and Tailoring of Implementation Strategies. J Behav Health Serv Res. 2017;44:177–94. 26289563 10.1007/s11414-015-9475-6 PMC4761530

[46] Powell BJ, Fernandez ME, Williams NJ, Aarons GA, Beidas RS, Lewis CC, et al. Enhancing the Impact of Implementation Strategies in Healthcare: A Research Agenda. Front Public Health [Internet]. 2019 [cited 2021 Mar 31];7. Available from: https://www.frontiersin.org/articles/10.3389/fpubh.2019.00003/full 10.3389/fpubh.2019.00003 PMC6350272 30723713

[47] Frakt AB, Prentice JC, Pizer SD, Elwy AR, Garrido MM, Kilbourne AM, et al. Overcoming Challenges to Evidence-Based Policy Development in a Large. Integrated Delivery System Health Serv Res. 2018;53:4789–807. 29862494 10.1111/1475-6773.12986 PMC6232400

[48] Crable EL, Lengnick-Hall R, Stadnick NA, Moullin JC, Aarons GA. Where is “policy” in dissemination and implementation science? Recommendations to advance theories, models, and frameworks: EPIS as a case example. Implement Sci. 2022;17:80. 36503520 10.1186/s13012-022-01256-x PMC9742035

[49] Crable EL, Grogan CM, Purtle J, Roesch SC, Aarons GA. Tailoring dissemination strategies to increase evidence-informed policymaking for opioid use disorder treatment: study protocol. Implement Sci Commun. 2023;4:16. 36797794 10.1186/s43058-023-00396-5 PMC9936679

[50] Bond GR. Evidence-based policy strategies: A typology. Clin Psychol Sci Pract. 2018;25:e12267. 10.1111/cpsp.12267

[51] Loo TS, Davis RB, Lipsitz LA, Irish J, Bates CK, Agarwal K, et al. Electronic Medical Record Reminders and Panel Management to Improve Primary Care of Elderly Patients. Arch Intern Med. 2011;171:1552–8. 21949163 10.1001/archinternmed.2011.394

[52] Shojania KG, Jennings A, Mayhew A, Ramsay C, Eccles M, Grimshaw J. Effect of point-of-care computer reminders on physician behaviour: a systematic review. CMAJ Can Med Assoc J. 2010;182:E216-25. 20212028 10.1503/cmaj.090578 PMC2842864

[53] Sequist TD, Gandhi TK, Karson AS, Fiskio JM, Bugbee D, Sperling M, et al. A Randomized Trial of Electronic Clinical Reminders to Improve Quality of Care for Diabetes and Coronary Artery Disease. J Am Med Inform Assoc JAMIA. 2005;12:431–7. 15802479 10.1197/jamia.M1788 PMC1174888

[54] Dopp AR, Kerns SEU, Panattoni L, Ringel JS, Eisenberg D, Powell BJ, et al. Translating economic evaluations into financing strategies for implementing evidence-based practices. Implement Sci. 2021;16:1–12. 34187520 10.1186/s13012-021-01137-9 PMC8240424

[55] Dopp AR, Hunter SB, Godley MD, Pham C, Han B, Smart R, et al. Comparing two federal financing strategies on penetration and sustainment of the adolescent community reinforcement approach for substance use disorders: protocol for a mixed-method study. Implement Sci Commun. 2022;3:51. 35562836 10.1186/s43058-022-00298-y PMC9099033

[56] Proctor EK, Toker E, Tabak R, McKay VR, Hooley C, Evanoff B. Market viability: a neglected concept in implementation science. Implement Sci. 2021;16:98. 34801036 10.1186/s13012-021-01168-2 PMC8605560

[57] Dopp AR, Narcisse M-R, Mundey P, Silovsky JF, Smith AB, Mandell D, et al. A scoping review of strategies for financing the implementation of evidence-based practices in behavioral health systems: State of the literature and future directions. Implement Res Pract. 2020;1:2633489520939980. 37089129 10.1177/2633489520939980 PMC9924261

[58] Dopp AR, Kerns SEU, Panattoni L, Ringel JS, Eisenberg D, Powell BJ, et al. Translating economic evaluations into financing strategies for implementing evidence-based practices. Implement Sci IS. 2021;16:66. 34187520 10.1186/s13012-021-01137-9 PMC8240424

[59] Kilbourne AM, Neumann MS, Pincus HA, Bauer MS, Stall R. Implementing evidence-based interventions in health care:application of the replicating effective programs framework. Implement Sci. 2007;2:42–51. 18067681 10.1186/1748-5908-2-42 PMC2248206

[60] Kegeles SM, Rebchook GM, Hays RB, Terry MA, O’Donnell L, Leonard NR, et al. From science to application: the development of an intervention package. AIDS Educ Prev Off Publ Int Soc AIDS Educ. 2000;12:62–74. 11063070

[61] Wandersman A, Imm P, Chinman M, Kaftarian S. Getting to outcomes: a results-based approach to accountability. Eval Program Plann. 2000;23:389–95. 10.1016/S0149-7189(00)00028-8

[62] Wandersman A, Chien VH, Katz J. Toward an evidence-based system for innovation support for implementing innovations with quality: Tools, training, technical assistance, and quality assurance/quality improvement. Am J Community Psychol. 2012;50:445–59. 22538406 10.1007/s10464-012-9509-7

[63] Rogal SS, Yakovchenko V, Waltz TJ, Powell BJ, Kirchner JE, Proctor EK, et al. The association between implementation strategy use and the uptake of hepatitis C treatment in a national sample. Implement Sci. 2017;12:1–13. 28494811 10.1186/s13012-017-0588-6 PMC5425997

[64] Smith SN, Almirall D, Prenovost K, Liebrecht C, Kyle J, Eisenberg D, et al. Change in patient outcomes after augmenting a low-level implementation strategy in community practices that are slow to adopt a collaborative chronic care model: a cluster randomized implementation trial. Med Care. 2019;57:503. 31135692 10.1097/MLR.0000000000001138 PMC6684247

[65] Rogal SS, Yakovchenko V, Waltz TJ, Powell BJ, Gonzalez R, Park A, et al. Longitudinal assessment of the association between implementation strategy use and the uptake of hepatitis C treatment: Year 2. Implement Sci. 2019;14:1–12. 30961615 10.1186/s13012-019-0881-7 PMC6454775

[66] Harvey G, Kitson A. Translating evidence into healthcare policy and practice: Single versus multi-faceted implementation strategies – is there a simple answer to a complex question? Int J Health Policy Manag. 2015;4:123–6. 25774368 10.15171/ijhpm.2015.54 PMC4357977

[67] Engell T, Stadnick NA, Aarons GA, Barnett ML. Common Elements Approaches to Implementation Research and Practice: Methods and Integration with Intervention Science. Glob Implement Res Appl. 2023;3:1–15. 37013068 10.1007/s43477-023-00077-4 PMC10063479

[68] Michie S, Fixsen D, Grimshaw JM, Eccles MP. Specifying and reporting complex behaviour change interventions: the need for a scientific method. Implement Sci IS. 2009;4:40. 19607700 10.1186/1748-5908-4-40 PMC2717906

[69] Smith JD, Li DH, Rafferty MR. The Implementation Research Logic Model: a method for planning, executing, reporting, and synthesizing implementation projects. Implement Sci IS. 2020;15:84. 32988389 10.1186/s13012-020-01041-8 PMC7523057

[70] Perez Jolles M, Lengnick-Hall R, Mittman BS. Core Functions and Forms of Complex Health Interventions: a Patient-Centered Medical Home Illustration. JGIM J Gen Intern Med. 2019;34:1032–8. 30623387 10.1007/s11606-018-4818-7 PMC6544719

[71] Schroeck FR, Ould Ismail AA, Haggstrom DA, Sanchez SL, Walker DR, Zubkoff L. Data-driven approach to implementation mapping for the selection of implementation strategies: a case example for risk-aligned bladder cancer surveillance. Implement Sci IS. 2022;17:58. 36050742 10.1186/s13012-022-01231-6 PMC9438061

[72] Frank HE, Kemp J, Benito KG, Freeman JB. Precision Implementation: An Approach to Mechanism Testing in Implementation Research. Adm Policy Ment Health. 2022;49:1084–94. 36167942 10.1007/s10488-022-01218-x

[73] Lewis CC, Klasnja P, Lyon AR, Powell BJ, Lengnick-Hall R, Buchanan G, et al. The mechanics of implementation strategies and measures: advancing the study of implementation mechanisms. Implement Sci Commun. 2022;3:114. 36273224 10.1186/s43058-022-00358-3 PMC9588220

[74] Geng EH, Baumann AA, Powell BJ. Mechanism mapping to advance research on implementation strategies. PLoS Med. 2022;19:e1003918. 35134069 10.1371/journal.pmed.1003918 PMC8824331

[75] Pinnock H, Barwick M, Carpenter CR, Eldridge S, Grandes G, Griffiths CJ, et al. Standards for Reporting Implementation Studies (StaRI) Statement. BMJ. 2017;356:i6795. 28264797 10.1136/bmj.i6795 PMC5421438

[76] Proctor E, Silmere H, Raghavan R, Hovmand P, Aarons G, Bunger A, et al. Outcomes for Implementation Research: Conceptual Distinctions, Measurement Challenges, and Research Agenda. Adm Policy Ment Health Ment Health Serv Res. 2011;38:65–76. 10.1007/s10488-010-0319-7 PMC3068522 20957426

[77] Hooley C, Amano T, Markovitz L, Yaeger L, Proctor E. Assessing implementation strategy reporting in the mental health literature: a narrative review. Adm Policy Ment Health Ment Health Serv Res. 2020;47:19–35. 10.1007/s10488-019-00965-8 PMC6946851 31482489

[78] Proctor E, Ramsey AT, Saldana L, Maddox TM, Chambers DA, Brownson RC. FAST: a framework to assess speed of translation of health innovations to practice and policy. Glob Implement Res Appl. 2022;2:107–19. 35669171 10.1007/s43477-022-00045-4 PMC9161655

[79] Cullen L, Hanrahan K, Edmonds SW, Reisinger HS, Wagner M. Iowa Implementation for Sustainability Framework. Implement Sci IS. 2022;17:1. 34983585 10.1186/s13012-021-01157-5 PMC8725573

[80] Saldana L, Ritzwoller DP, Campbell M, Block EP. Using economic evaluations in implementation science to increase transparency in costs and outcomes for organizational decision-makers. Implement Sci Commun. 2022;3:40. 35410434 10.1186/s43058-022-00295-1 PMC9004101

[81] Eisman AB, Kilbourne AM, Dopp AR, Saldana L, Eisenberg D. Economic evaluation in implementation science: making the business case for implementation strategies. Psychiatry Res. 2020;283:112433. 31202612 10.1016/j.psychres.2019.06.008 PMC6898762

[82] Akiba CF, Powell BJ, Pence BW, Nguyen MX, Golin C, Go V. The case for prioritizing implementation strategy fidelity measurement: benefits and challenges. Transl Behav Med. 2022;12:335–42. 34791480 10.1093/tbm/ibab138 PMC8849000

[83] Akiba CF, Powell BJ, Pence BW, Muessig K, Golin CE, Go V. “We start where we are”: a qualitative study of barriers and pragmatic solutions to the assessment and reporting of implementation strategy fidelity. Implement Sci Commun. 2022;3:117. 36309715 10.1186/s43058-022-00365-4 PMC9617230

[84] Rudd BN, Davis M, Doupnik S, Ordorica C, Marcus SC, Beidas RS. Implementation strategies used and reported in brief suicide prevention intervention studies. JAMA Psychiatry. 2022;79:829–31. 35704303 10.1001/jamapsychiatry.2022.1462 PMC9201740

[85] Painter JT, Raciborski RA, Matthieu MM, Oliver CM, Adkins DA, Garner KK. Engaging stakeholders to retrospectively discern implementation strategies to support program evaluation: Proposed method and case study. Eval Program Plann. 2024;103:102398. 10.1016/j.evalprogplan.2023.102398 38183893

[86] Bunger AC, Powell BJ, Robertson HA, MacDowell H, Birken SA, Shea C. Tracking implementation strategies: a description of a practical approach and early findings. Health Res Policy Syst. 2017;15:1–12. 28231801 10.1186/s12961-017-0175-y PMC5324332

[87] Mustanski B, Smith JD, Keiser B, Li DH, Benbow N. Supporting the growth of domestic HIV implementation research in the united states through coordination, consultation, and collaboration: how we got here and where we are headed. JAIDS J Acquir Immune Defic Syndr. 2022;90:S1-8. 35703749 10.1097/QAI.0000000000002959 PMC9643076

[88] Marques MM, Wright AJ, Corker E, Johnston M, West R, Hastings J, et al. The Behaviour Change Technique Ontology: Transforming the Behaviour Change Technique Taxonomy v1. Wellcome Open Res. 2023;8:308. 37593567 10.12688/wellcomeopenres.19363.1 PMC10427801

[89] Merle JL, Li D, Keiser B, Zamantakis A, Queiroz A, Gallo CG, et al. Categorising implementation determinants and strategies within the US HIV implementation literature: a systematic review protocol. BMJ Open. 2023;13:e070216. 36927593 10.1136/bmjopen-2022-070216 PMC10030793

[90] Glenshaw MT, Gaist P, Wilson A, Cregg RC, Holtz TH, Goodenow MM. Role of NIH in the Ending the HIV Epidemic in the US Initiative: Research Improving Practice. J Acquir Immune Defic Syndr. 1999;2022(90):S9-16. 10.1097/QAI.0000000000002960 35703750

[91] Purcell DW, Namkung Lee A, Dempsey A, Gordon C. Enhanced Federal Collaborations in Implementation Science and Research of HIV Prevention and Treatment. J Acquir Immune Defic Syndr. 1999;2022(90):S17-22. 10.1097/QAI.0000000000002961 35703751

[92] Queiroz A, Mongrella M, Keiser B, Li DH, Benbow N, Mustanski B. Profile of the Portfolio of NIH-Funded HIV Implementation Research Projects to Inform Ending the HIV Epidemic Strategies. J Acquir Immune Defic Syndr. 1999;2022(90):S23-31. 10.1097/QAI.0000000000002962 PMC10204808 35703752

[93] Zamantakis A, Li DH, Benbow N, Smith JD, Mustanski B. Determinants of Pre-exposure Prophylaxis (PrEP) Implementation in Transgender Populations: A Qualitative Scoping Review. AIDS Behav. 2023;27:1600–18. 36520334 10.1007/s10461-022-03943-8 PMC9753072

[94] Li DH, Benbow N, Keiser B, Mongrella M, Ortiz K, Villamar J, et al. Determinants of Implementation for HIV Pre-exposure Prophylaxis Based on an Updated Consolidated Framework for Implementation Research: A Systematic Review. J Acquir Immune Defic Syndr. 1999;2022(90):S235-46. 10.1097/QAI.0000000000002984 PMC10161203 35703776

[95] Chambers DA, Emmons KM. Navigating the field of implementation science towards maturity: challenges and opportunities. Implement Sci. 2024;19:26, s13012-024-01352–0. 10.1186/s13012-024-01352-0 PMC10936041 38481286

[96] Chinman M, Acosta J, Ebener P, Shearer A. “What we have here, is a failure to [replicate]”: Ways to solve a replication crisis in implementation science. Prev Sci. 2022;23:739–50. 34312769 10.1007/s11121-021-01286-9 PMC12884401

[97] Chambers DA, Glasgow RE, Stange KC. The dynamic sustainability framework: addressing the paradox of sustainment amid ongoing change. Implement Sci. 2013;8:117. 24088228 10.1186/1748-5908-8-117 PMC3852739

[98] Lengnick-Hall R, Gerke DR, Proctor EK, Bunger AC, Phillips RJ, Martin JK, et al. Six practical recommendations for improved implementation outcomes reporting. Implement Sci. 2022;17:16. 35135566 10.1186/s13012-021-01183-3 PMC8822722

[99] Miller CJ, Barnett ML, Baumann AA, Gutner CA, Wiltsey-Stirman S. The FRAME-IS: a framework for documenting modifications to implementation strategies in healthcare. Implement Sci IS. 2021;16:36. 33827716 10.1186/s13012-021-01105-3 PMC8024675

[100] Xu X, Lazar CM, Ruger JP. Micro-costing in health and medicine: a critical appraisal. Health Econ Rev. 2021;11:1. 33404857 10.1186/s13561-020-00298-5 PMC7789519

[101] Barnett ML, Dopp AR, Klein C, Ettner SL, Powell BJ, Saldana L. Collaborating with health economists to advance implementation science: a qualitative study. Implement Sci Commun. 2020;1:82. 33005901 10.1186/s43058-020-00074-w PMC7523377

[102] Lengnick-Hall R, Williams NJ, Ehrhart MG, Willging CE, Bunger AC, Beidas RS, et al. Eight characteristics of rigorous multilevel implementation research: a step-by-step guide. Implement Sci. 2023;18:52. 37872618 10.1186/s13012-023-01302-2 PMC10594828

[103] Riley-Gibson E, Hall A, Shoesmith A, Wolfenden L, Shelton RC, Doherty E, et al. A systematic review to determine the effect of strategies to sustain chronic disease prevention interventions in clinical and community settings: study protocol. Res Sq [Internet]. 2023 [cited 2024 Apr 19]; Available from: https://www.ncbi.nlm.nih.gov/pmc/articles/PMC10312971/10.1186/s13643-024-02541-0 PMC11084058 38725053

[104] Ingvarsson S, Hasson H, von Thiele Schwarz U, Nilsen P, Powell BJ, Lindberg C, et al. Strategies for de-implementation of low-value care—a scoping review. Implement Sci IS. 2022;17:73. 36303219 10.1186/s13012-022-01247-y PMC9615304

[105] Lewis CC, Powell BJ, Brewer SK, Nguyen AM, Schriger SH, Vejnoska SF, et al. Advancing mechanisms of implementation to accelerate sustainable evidence-based practice integration: protocol for generating a research agenda. BMJ Open. 2021;11:e053474. 34663668 10.1136/bmjopen-2021-053474 PMC8524292

[106] Hailemariam M, Bustos T, Montgomery B, Barajas R, Evans LB, Drahota A. Evidence-based intervention sustainability strategies: a systematic review. Implement Sci. 2019;14:N.PAG-N.PAG. 10.1186/s13012-019-0910-6 PMC6554955 31171004

[107] Michie S, Atkins L, West R. The behaviour change wheel. Guide Des Interv 1st Ed G B Silverback Publ. 2014;1003:1010.

[108] Birken SA, Haines ER, Hwang S, Chambers DA, Bunger AC, Nilsen P. Advancing understanding and identifying strategies for sustaining evidence-based practices: a review of reviews. Implement Sci IS. 2020;15:88. 33036653 10.1186/s13012-020-01040-9 PMC7545853

[109] Metz A, Jensen T, Farley A, Boaz A, Bartley L, Villodas M. Building trusting relationships to support implementation: A proposed theoretical model. Front Health Serv. 2022;2:894599. 36925800 10.3389/frhs.2022.894599 PMC10012819

[110] Rabin BA, Cain KL, Watson P, Oswald W, Laurent LC, Meadows AR, et al. Scaling and sustaining COVID-19 vaccination through meaningful community engagement and care coordination for underserved communities: hybrid type 3 effectiveness-implementation sequential multiple assignment randomized trial. Implement Sci IS. 2023;18:28. 37443044 10.1186/s13012-023-01283-2 PMC10347705

[111] Gyamfi J, Iwelunmor J, Patel S, Irazola V, Aifah A, Rakhra A, et al. Implementation outcomes and strategies for delivering evidence-based hypertension interventions in lower-middle-income countries: Evidence from a multi-country consortium for hypertension control. PLOS ONE. 2023;18:e0286204. 37228144 10.1371/journal.pone.0286204 PMC10212179

[112] Woodward EN, Ball IA, Willging C, Singh RS, Scanlon C, Cluck D, et al. Increasing consumer engagement: tools to engage service users in quality improvement or implementation efforts. Front Health Serv. 2023;3:1124290. 37560195 10.3389/frhs.2023.1124290 PMC10407803

[113] Norton WE, Chambers DA. Unpacking the complexities of de-implementing inappropriate health interventions. Implement Sci IS. 2020;15:2. 31915032 10.1186/s13012-019-0960-9 PMC6950868

[114] Norton WE, McCaskill-Stevens W, Chambers DA, Stella PJ, Brawley OW, Kramer BS. DeImplementing Ineffective and Low-Value Clinical Practices: Research and Practice Opportunities in Community Oncology Settings. JNCI Cancer Spectr. 2021;5:pkab020. 33860151 10.1093/jncics/pkab020 PMC8036997

[115] McKay VR, Proctor EK, Morshed AB, Brownson RC, Prusaczyk B. Letting Go: Conceptualizing Intervention De-implementation in Public Health and Social Service Settings. Am J Community Psychol. 2018;62:189–202. 29971792 10.1002/ajcp.12258 PMC6175194

[116] Patey AM, Grimshaw JM, Francis JJ. Changing behaviour, ‘more or less’: do implementation and de-implementation interventions include different behaviour change techniques? Implement Sci IS. 2021;16:20. 33632274 10.1186/s13012-021-01089-0 PMC7905859

[117] Rodriguez Weno E, Allen P, Mazzucca S, Farah Saliba L, Padek M, Moreland-Russell S, et al. Approaches for Ending Ineffective Programs: Strategies From State Public Health Practitioners. Front Public Health. 2021;9:727005. 34490203 10.3389/fpubh.2021.727005 PMC8417719

[118] Gnjidic D, Elshaug AG. De-adoption and its 43 related terms: harmonizing low-value care terminology. BMC Med. 2015;13:273. 26486727 10.1186/s12916-015-0511-4 PMC4617953

