# Publisher Correction: A systematic review of experimentally tested implementation strategies across health and human service settings: evidence from 2010-2022

**DOI:** 10.1186/s13012-024-01377-5

**Published:** 2024-07-24

**Authors:** Laura Ellen Ashcraft, David E. Goodrich, Joachim Hero, Angela Phares, Rachel L. Bachrach, Deirdre A. Quinn, Nabeel Qureshi, Natalie C. Ernecof, Lisa G. Lederer, Leslie Page Scheunemann, Shari S. Rogal, Matthew J. Chinman

**Affiliations:** 1 Center for Health Equity Research and Promotion, Corporal Michael Crescenz VA Medical Center, Philadelphia, PA USA; 2 Department of Biostatistics, Epidemiology, and Informatics, University of Pennsylvania Perelman School of Medicine, Philadelphia, PA USA; 3 Center for Health Equity Research and Promotion, VA Pittsburgh Healthcare System, Pittsburgh, PA USA; 4 Division of General Internal Medicine, Department of Medicine, University of Pittsburgh, Pittsburgh, PA USA; 5 Clinical & Translational Science Institute, University of Pittsburgh, Pittsburgh, PA USA; 6 RAND Corporation, Pittsburgh, PA USA; 7 Center for Clinical Management Research, VA Ann Arbor Healthcare System, Ann Arbor, MI USA; 8 Department of Psychiatry, University of Michigan Medical School, Ann Arbor, MI USA; 9 Division of Geriatric Medicine, University of Pittsburgh, Department of Medicine, Pittsburgh, PA USA; 10 Division of Pulmonary, Allergy, Critical Care, and Sleep Medicine, University of Pittsburgh, Department of Medicine, Pittsburgh, PA USA; 11 Departments of Medicine and Surgery, University of Pittsburgh, Pittsburgh, PA USA

**Correction** **:** **Implement Sci 19, 43 (2024)**

**https://doi.org/10.1186/s13012-024-01369-5**

The Original Article [1] contained incorrect Supplementary Materials due to typesetting errors. Several irrelevant files, including a cover letter and a manuscript draft, were mistakenly processed as Supplementary Materials.

The correct Supplementary Materials are included with this Correction.

The Original Article has been corrected. The publisher apologizes for the inconvenience caused to the authors.

### Supplementary Information

Supplementary file 1.

Supplementary file 2.

Supplementary file 3.

Supplementary file 4.

Supplementary file 5.
