# Supplementary material for: A systematic review of experimentally tested implementation strategies across health and human service settings: evidence from 2010-2022
Source: Implement Sci. 2024 Jun 24;19:43. doi: 10.1186/s13012-024-01369-5 (PMC11194895; doi:10.1186/s13012-024-01369-5)
Supplement: Supplementary file 2 — Supplementary Material 2. [file 13012_2024_1369_MOESM2_ESM.docx]

**Databases:** PubMed and CINAHL

Fields: Title or Abstract fields

Language: English

Dates 2010-2022

1. "implementation strateg*" OR "implementation interventio*" OR "implementation bundl*" OR "implementation support*"

**Database:** Web of Science

Language: English

Dates: 2010-2022

Indexes: SCI-EXPANDED, SSCI, A&HCI, ESCI.

1. **TOPIC:**("implementation strategies") OR **TOPIC:** ("implementation strategy") OR **TOPIC:**("implementation intervention") OR **TOPIC:** ("implementation interventions") OR **TOPIC:** ("implementation bundles") OR **TOPIC:** ("implementation bundle") OR **TOPIC:** ("implementation support") OR **TOPIC:**("implementation supports")
