# Supplementary material for: A systematic review of experimentally tested implementation strategies across health and human service settings: evidence from 2010-2022
Source: Implement Sci. 2024 Jun 24;19:43. doi: 10.1186/s13012-024-01369-5 (PMC11194895; doi:10.1186/s13012-024-01369-5)
Supplement: Supplementary file 3 — Supplementary Material 3. [file 13012_2024_1369_MOESM3_ESM.docx]

**Additional File 3. Systematic reviews examined for eligible studies**

*Reviews are in alphabetical order by first author last name.*

| **Publication Year** | **Authors** | **Title** | **Journal** |
| --- | --- | --- | --- |
| 2021 | A. Al-Jawaldeh; M. Taktouk; A. Chatila; S. Naalbandian; A. M. Al-Thani; M. M. Alkhalaf; S. Almamary; R. Barham; N. M. Baqadir; F. F. Binsunaid; G. Fouad; L. Nasreddine | Salt Reduction Initiatives in the Eastern Mediterranean Region and Evaluation of Progress towards the 2025 Global Target: A Systematic Review | Nutrients |
| 2022 | A. Alishahi Tabriz; K. Turner; A. Clary; Y. R. Hong; O. T. Nguyen; G. Wei; R. B. Carlson; S. A. Birken | De-implementing low-value care in cancer care delivery: a systematic review | Implement Sci |
| 2021 | A. Losada-Baltar; L. Jiménez-Gonzalo | Translating evidence into practice: nonpharmacological interventions for BPSD | International Psychogeriatrics |
| 2021 | A. M. Patey; J. M. Grimshaw; J. J. Francis | Changing behaviour, 'more or less': do implementation and de-implementation interventions include different behaviour change techniques? | Implement Sci |
| 2022 | A. Mullen; G. Browne; B. Hamilton; S. Skinner; B. Happell | Safewards: An integrative review of the literature within inpatient and forensic mental health units | International journal of mental health nursing |
| 2022 | A. Oral | Are implementation interventions effective in promoting the adoption of evidence-based practices in stroke rehabilitation? A Cochrane Review summary with commentary | Neurorehabilitation |
| 2022 | A. Ugalde; V. White; N. M. Rankin; C. Paul; C. Segan; S. Aranda; A. Wong Shee; A. M. Hutchinson; P. M. Livingston | How can hospitals change practice to better implement smoking cessation interventions? A systematic review | Ca Cancer J Clin |
| 2019 | Abdallah, A. B.; Alkhaldi, R. Z. | Lean bundles in health care: a scoping review | J Health Organ Manag |
| 2019 | Ariyo, Promise; Zayed, Bassem; Riese, Victoria; Anton, Blair; Latif, Asad; Kilpatrick, Claire; Allegranzi, Benedetta; Berenholtz, Sean | Implementation strategies to reduce surgical site infections: A systematic review | Infection Control And Hospital Epidemiology |
| 2022 | B. Bovendeerd; K. De Jong; E. De Groot; M. Moerbeek; J. De Keijser | Enhancing the effect of psychotherapy through systematic client feedback in outpatient mental healthcare: A cluster randomized trial | Psychother Res |
| 2021 | B. Prediger; A. Heu-Parvaresch; S. Polus; S. Bühn; E. A. M. Neugebauer; P. Dawid | A systematic review on the effectiveness of implementation strategies to postpone elective caesarean sections to ≥ 39 + (0-6) weeks of gestation | Syst Rev |
| 2019 | Barrera-Cancedda, Amy Elizabeth; Riman, Kathryn A.; Shinnick, Julianna E.; Buttenheim, Alison M. | Implementation strategies for infection prevention and control promotion for nurses in Sub-Saharan Africa: a systematic review | Implementation Science |
| 2019 | Beauchemin, Melissa; Murray, Meghan T.; Sung, Lillian; Hershman, Dawn L.; Weng, Chunhua; Schnall, Rebecca | Clinical decision support for therapeutic decision-making in cancer: A systematic review | International Journal Of Medical Informatics |
| 2019 | Bird, M. L.; Miller, T.; Connell, L. A.; Eng, J. J. | Moving stroke rehabilitation evidence into practice: a systematic review of randomized controlled trials | Clinical Rehabilitation |
| 2019 | Bunting, Lisa; Montgomery, Lorna; Mooney, Suzanne; MacDonald, Mandi; Coulter, Stephen; Hayes, David; Davidson, Gavin | Trauma Informed Child Welfare Systems—A Rapid Evidence Review. | International Journal Of Environmental Research And Public Health |
| 2022 | C. B. Uwizeye; H. T. V. Zomahoun; A. Bussières; A. Thomas; D. Kairy; J. Massougbodji; N. Rheault; S. Tchoubi; L. Philibert; S. Abib Gaye; L. Khadraoui; A. Ben Charif; E. Diendéré; L. Langlois; M. Dugas; F. Légaré | Implementation Strategies for Knowledge Products in Primary Health Care: Systematic Review of Systematic Reviews | Interact J Med Res |
| 2021 | C. C. Orelio; P. Heus; J. J. Kroese-van Dieren; R. Spijker; B. C. van Munster; L. Hooft | Reducing Inappropriate Proton Pump Inhibitors Use for Stress Ulcer Prophylaxis in Hospitalized Patients: Systematic Review of De-Implementation Studies | J Gen Intern Med |
| 2021 | C. E. Cassidy; M. B. Harrison; C. Godfrey; V. Nincic; P. A. Khan; P. Oakley; A. Ross-White; H. Grantmyre; I. D. Graham | Use and effects of implementation strategies for practice guidelines in nursing: a systematic review | Implement Sci |
| 2022 | C. M. Stoney; M. K. Brown; R. A. Campo; G. A. Mensah | Integration of implementation science in cardiovascular behavioral medicine | Health Psychol |
| 2020 | Cahill, Liana S.; Carey, Leeanne M.; Lannin, Natasha A.; Turville, Megan; Neilson, Cheryl L.; Lynch, Elizabeth A.; McKinstry, Carol E.; Han, Jia Xi; O'Connor, Denise | Implementation interventions to promote the uptake of evidence-based practices in stroke rehabilitation | Cochrane Database Of Systematic Reviews |
| 2019 | Cancelliere, Carol; Sutton, Deborah; Côté, Pierre; French, Simon D.; Taylor-Vaisey, Anne; Mior, Silvano A. | Implementation interventions for musculoskeletal programs of care in the active military and barriers, facilitators, and outcomes of implementation: a scoping review | Implementation Science |
| 2021 | D. E. Winchester; J. Merritt; N. Waheed; H. Norton; V. Manja; N. R. Shah; C. D. Helfrich | Implementation of appropriate use criteria for cardiology tests and procedures: a systematic review and meta-analysis | Eur Heart J Qual Care Clin Outcomes |
| 2022 | D. L. Belavy; S. D. Tagliaferri; P. Buntine; T. Saueressig; C. Samanna; T. McGuckian; C. T. Miller; P. J. Owen | Reducing Low-Value Imaging for Low Back Pain: Systematic Review With Meta-analysis | Journal Of Orthopaedic & Sports Physical Therapy |
| 2022 | D. L. Belavy; S. D. Tagliaferri; P. Buntine; T. Saueressig; K. Sadler; C. Ko; C. T. Miller; P. J. Owen | Clinician education unlikely effective for guideline-adherent medication prescription in low back pain: systematic review and meta-analysis of RCTs | Eclinicalmedicine |
| 2021 | D. Sud; E. Laughton; R. McAskill; E. Bradley; I. Maidment | The role of pharmacy in the management of cardiometabolic risk, metabolic syndrome and related diseases in severe mental illness: a mixed-methods systematic literature review | Syst Rev |
| 2021 | E. Louie; E. L. Barrett; A. Baillie; P. Haber; K. C. Morley | A systematic review of evidence-based practice implementation in drug and alcohol settings: applying the consolidated framework for implementation research framework | Implement Sci |
| 2018 | Ebben, Remco H. A.; Siqeca, Flaka; Madsen, Ulla Riis; Vloet, Lilian C. M.; van Achterberg, Theo | Effectiveness of implementation strategies for the improvement of guideline and protocol adherence in emergency care: a systematic review | Bmj Open |
| 2018 | Finley, Cara; Suellentrop, Katherine; Griesse, Rebecca; House, Lawrence Duane; Brittain, Anna | Stakeholder Education for Community-Wide Health Initiatives: A Focus on Teen Pregnancy Prevention | Health Promotion Practice |
| 2019 | Flodgren, G.; Brien, M. A.; Parmelli, E.; Grimshaw, J. M. | Local opinion leaders: effects on professional practice and healthcare outcomes | Cochrane Database Of Systematic Reviews |
| 2021 | G. Neta; M. Clyne; D. A. Chambers | Dissemination and Implementation Research at the National Cancer Institute: A Review of Funded Studies (2006-2019) and Opportunities to Advance the Field | Cancer Epidemiol Biomarkers Prev |
| 2018 | Glidewell, Liz; Willis, Thomas A.; Petty, Duncan; Lawton, Rebecca; McEachan, Rosemary R. C.; Ingleson, Emma; Heudtlass, Peter; Davies, Andrew; Jamieson, Tony; Hunter, Cheryl; Hartley, Suzanne; Gray-Burrows, Kara; Clamp, Susan; Carder, Paul; Alderson, Sarah; Farrin, Amanda J.; Foy, Robbie; on behalf of the, Aspire programme team; team, Aspire programme | To what extent can behaviour change techniques be identified within an adaptable implementation package for primary care? A prospective directed content analysis | Implementation Science |
| 2021 | H. Augustsson; S. Ingvarsson; P. Nilsen; U. von Thiele Schwarz; I. Muli; J. Dervish; H. Hasson | Determinants for the use and de-implementation of low-value care in health care: a scoping review | Implement Sci Commun |
| 2022 | H. Grønningsæter; C. Kiland | Theory-based implementation of physical activity programs in worksite health promotion settings: a systematic review | Health Promotion International |
| 2019 | Hailemariam, M., Bustos, T., Montgomery, B. | Evidence-based intervention sustainability strategies: a systematic review | Implementation Sci |
| 2021 | I. Mushamiri; W. Belai; E. Sacks; B. Genberg; S. Gupta; H. B. Perry | Evidence on the effectiveness of community-based primary health care in improving HIV/AIDS outcomes for mothers and children in low- and middle-income countries: Findings from a systematic review | J Glob Health |
| 2018 | Ijsbrandy, C.; Ottevanger, P. B.; Tsekou Diogeni, M.; Gerritsen, W. R.; van Harten, W. H.; Hermens, R. P. M. G. | Review: Effectiveness of implementation strategies to increase physical activity uptake during and after cancer treatment | Critical Reviews In Oncology/Hematology |
| 2018 | Johnson, Lauren G.; Armstrong, Allison; Joyce, Caroline M.; Teitelman, Anne M.; Buttenheim, Alison M. | Implementation Strategies to Enhance the Implementation of eHealth Programs for Patients With Chronic Illnesses: Realist Systematic Review | Implementation Science |
| 2022 | K. Adhikari; K. Manalili; J. Law; M. Bischoff; G. F. Teare | Interventions to Increase Colorectal Cancer Screening Uptake in Primary Care: A Systematic Review | J Am Board Fam Med |
| 2021 | K. S. Dickson; T. Lind; A. Jobin; M. Kinnear; H. Lok; L. Brookman-Frazee | A Systematic Review of Mental Health Interventions for ASD: Characterizing Interventions, Intervention Adaptations, and Implementation Outcomes | Adm Policy Ment Health |
| 2020 | Kerr, Justin; Devane, Declan; Ivory, John; Weller, Carolina; Gethin, Georgina | Effectiveness of implementation strategies for venous leg ulcer guidelines: A systematic review | Journal Of Tissue Viability |
| 2018 | Kovacs, Eva; Strobl, Ralf; Phillips, Amanda; Stephan, Anna-Janina; Müller, Martin; Gensichen, Jochen; Grill, Eva | Systematic Review and Meta-analysis of the Effectiveness of Implementation Strategies for Non-communicable Disease Guidelines in Primary Health Care | JGIM: Journal Of General Internal Medicine |
| 2022 | L. K. Jones; R. C. Brownson; M. S. Williams | Applying implementation science to improve care for familial hypercholesterolemia | Curr Opin Endocrinol Diabetes Obes |
| 2021 | L. K. Jones; S. Tilberry; C. Gregor; L. H. Yaeger; Y. Hu; A. C. Sturm; T. L. Seaton; T. J. Waltz; A. K. Rahm; A. Goldberg; R. C. Brownson; S. S. Gidding; M. S. Williams; M. R. Gionfriddo | Implementation strategies to improve statin utilization in individuals with hypercholesterolemia: a systematic review and meta-analysis | Implement Sci |
| 2022 | L. Wolfenden; S. McCrabb; C. Barnes; K. M. O'Brien; K. W. Ng; N. K. Nathan; R. Sutherland; R. K. Hodder; F. Tzelepis; E. Nolan; C. M. Williams; S. L. Yoong | Strategies for enhancing the implementation of school-based policies or practices targeting diet, physical activity, obesity, tobacco or alcohol use | Cochrane Database Syst Rev |
| 2021 | M. C. White; K. Peven; O. Clancy; I. Okonkwo; I. Bakolis; S. Russ; A. J. M. Leather; N. Sevdalis | Implementation Strategies and the Uptake of the World Health Organization Surgical Safety Checklist in Low and Middle Income Countries: A Systematic Review and Meta-analysis | Ann Surg |
| 2021 | M. de Foubert; H. Cummins; R. McCullagh; V. Brueton; C. Naughton | Systematic review of interventions targeting fundamental care to reduce hospital-associated decline in older patients | Journal of advanced nursing |
| 2021 | M. Neuman; K. L. Fielding; H. Ayles; F. M. Cowan; B. Hensen; P. P. Indravudh; C. Johnson; E. L. Sibanda; K. Hatzold; E. L. Corbett | ART initiations following community-based distribution of HIV self-tests: meta-analysis and meta-regression of STAR Initiative data | Bmj Glob Health |
| 2021 | M. T. Silva; T. F. Galvao; E. Chapman; E. N. da Silva; J. O. M. Barreto | Dissemination interventions to improve healthcare workers' adherence with infection prevention and control guidelines: a systematic review and meta-analysis | Implement Sci |
| 2020 | Marche, Benedikt; Neuwirth, Meike; Kugler, Christiane; Bouillon, Bertil; Mattner, Frauke; Otchwemah, Robin | Implementation methods of infection prevention measures in orthopedics and traumatology - a systematic review | European Journal Of Trauma And Emergency Surgery |
| 2018 | McFadyen, T.; Chai, L. K.; Wyse, R.; Kingsland, M.; Yoong, S. L.; Clinton-McHarg, T.; Bauman, A.; Wiggers, J.; Rissel, C.; Williams, C. M.; Wolfenden, L. | Strategies to improve the implementation of policies, practices or programmes in sporting organisations targeting poor diet, physical inactivity, obesity, risky alcohol use or tobacco use: a systematic review | Bmj Open |
| 2020 | Miller, Emily S.; Jensen, Rebekah; Hoffman, M. Camille; Osborne, Lauren M.; McEvoy, Katherine; Grote, Nancy; Moses-Kolko, Eydie L. | Implementation of perinatal collaborative care: a health services approach to perinatal depression care | Primary Health Care Research And Development |
| 2018 | Mills, Katherine T.; Obst, Katherine M.; Shen, Wei; Molina, Sandra; Zhang, Hui-Jie; He, Hua; Cooper, Lisa A.; He, Jiang | Comparative Effectiveness of Implementation Strategies for Blood Pressure Control in Hypertensive Patients: A Systematic Review and Meta-analysis | Annals Of Internal Medicine |
| 2019 | Munar, Wolfgang; Snilstveit, Birte; Aranda, Ligia Esther; Biswas, Nilakshi; Baffour, Theresa; Stevenson, Jenniffer | Evidence gap map of performance measurement and management in primary healthcare systems in low-income and middle-income countries. | Bmj Global Health |
| 2021 | P. D. Franklin; C. P. Bond; N. E. Rothrock; D. Cella | Strategies for Effective Implementation of Patient-Reported Outcome Measures in Arthroplasty Practice | J Bone Joint Surg Am |
| 2022 | P. S. L. da Silva; M. E. Reis; D. Farah; T. R. M. Andrade; M. C. M. Fonseca | Care bundles to reduce unplanned extubation in critically ill children: a systematic review, critical appraisal and meta-analysis | Arch Dis Child |
| 2020 | Peven, K.; Bick, D.; Purssell, E.; Rotevatn, T. A.; Nielsen, J. H.; Taylor, C. | Evaluating implementation strategies for essential newborn care interventions in low- and low middle-income countries: a systematic review | Health Policy Plan |
| 2018 | Phelan, Sonja; Lin, Frances; Mitchell, Marion; Chaboyer, Wendy | Implementing early mobilisation in the intensive care unit: An integrative review | International Journal Of Nursing Studies |
| 2018 | Phillips, Cameron J.; Wisdom, Alice J.; McKinnon, Ross A.; Woodman, Richard J.; Gordon, David L. | Interventions targeting the prescribing and monitoring of vancomycin for hospitalized patients: a systematic review with meta-analysis. | Infection And Drug Resistance |
| 2022 | R. Fillipo; K. Pruka; M. Carvalho; M. E. Horn; J. Moore; B. Ramger; D. Clewley | Does the implementation of clinical practice guidelines for low back and neck pain by physical therapists improve patient outcomes? A systematic review | Implement Sci Commun |
| 2020 | Rietbergen, Tessa; Spoon, Denise; Brunsveld-Reinders, Anja H.; Schoones, Jan W.; Huis, Anita; Heinen, Maud; Persoon, Anke; van Dijk, Monique; Vermeulen, Hester; Ista, Erwin; van Bodegom-Vos, Leti | Effects of de-implementation strategies aimed at reducing low-value nursing procedures: a systematic review and meta-analysis | Implementation Science |
| 2020 | Ryan, N.; Vieira, D.; Goffman, D.; Bloch, E. M.; Akaba, G. O.; D'Mello B, S.; Egekeze, C.; Snyder, A.; Lyimo, M.; Nnodu, O.; Peprah, E. | Implementation outcomes of policy and programme innovations to prevent obstetric haemorrhage in low- and middle-income countries: a systematic review | Health Policy Plan |
| 2022 | S. Ahuja; N. Peiffer-Smadja; K. Peven; M. White; A. J. M. Leather; S. Singh; M. Mendelson; A. Holmes; G. Birgand; N. Sevdalis | Use of Feedback Data to Reduce Surgical Site Infections and Optimize Antibiotic Use in Surgery: A Systematic Scoping Review | Ann Surg |
| 2021 | S. Bennett; K. Laver; M. MacAndrew; E. Beattie; L. Clemson; C. Runge; L. Richardson | Implementation of evidence-based, non-pharmacological interventions addressing behavior and psychological symptoms of dementia: a systematic review focused on implementation strategies | Int Psychogeriatr |
| 2021 | S. Liu; T. J. Reese; K. Kawamoto; G. Del Fiol; C. Weir | A theory-based meta-regression of factors influencing clinical decision support adoption and implementation | J Am Med Inform Assoc |
| 2018 | Shanbhag, Deeptj; Graham, Ian D.; Harlos, Karen; Haynes, R. Brian; Gabizon, Itzhak; Connolly, Stuart J.; Van Spall, Harriette Gillian Christine | Effectiveness of implementation interventions in improving physician adherence to guideline recommendations in heart failure: a systematic review | Bmj Open |
| 2020 | Spoon, Denise; Rietbergen, Tessa; Huis, Anita; Heinen, Maud; van Dijk, Monique; van Bodegom-Vos, Leti; Ista, Erwin | Implementation strategies used to implement nursing guidelines in daily practice: A systematic review | International Journal Of Nursing Studies |
| 2021 | T. A. Loughran; J. L. Scharer; L. Rodriguez; M. J. De Vita; S. A. Maisto; J. S. Funderburk | Brief alcohol interventions in U.S. medical settings: A systematic review of the implementation literature | J Subst Abuse Treat |
| 2022 | T. Amano; C. Hooley; J. Strong; M. Inoue | Strategies for implementing music-based interventions for people with dementia in long-term care facilities: A systematic review | International Journal of Geriatric Psychiatry |
| 2022 | T. L. Morgan; C. Romani; A. Ross-White; A. Latimer-Cheung; J. R. Tomasone | Dissemination and implementation strategies for physical activity guidelines among adults with disability, chronic conditions, and pregnancy: a systematic scoping review | Bmc Public Health |
| 2021 | T. Melton; H. Jasmin; H. F. Johnson; A. Coley; S. Duffey; C. P. Renfro | Describing the delivery of clinical pharmacy services via telehealth: A systematic review | Journal Of The American College Of Clinical Pharmacy |
| 2020 | Talevski, Jason; Shee, Anna Wong; Rasmussen, Bodil; Kemp, Georgie; Beauchamp, Alison | Teach-back: A systematic review of implementation and impacts | Plos One |
| 2020 | Tomasone, Jennifer R.; Kauffeldt, Kaitlyn D.; Chaudhary, Rushil; Brouwers, Melissa C. | Effectiveness of guideline dissemination and implementation strategies on health care professionals' behaviour and patient outcomes in the cancer care context: a systematic review | Implementation Science |
| 2022 | V. Seda; R. J. Moles; S. R. Carter; C. R. Schneider | Assessing the comparative effectiveness of implementation strategies for professional services to community pharmacy: A systematic review | Res Social Adm Pharm |
| 2019 | Varsi, C.; Solberg Nes, L.; Kristjansdottir, O. B.; Kelders, S. M.; Stenberg, U.; Zangi, H. A.; Børøsund, E.; Weiss, K. E.; Stubhaug, A.; Asbjørnsen, R. A.; Westeng, M.; Ødegaard, M.; Eide, H. | Implementation Strategies to Enhance the Implementation of eHealth Programs for Patients With Chronic Illnesses: Realist Systematic Review | J Med Internet Res |
| 2019 | Villarosa, Amy R.; Maneze, Della; Ramjan, Lucie M.; Srinivas, Ravi; Camilleri, Michelle; George, Ajesh | The effectiveness of guideline implementation strategies in the dental setting: a systematic review | Implementation Science |
| 2020 | Wagenaar, Bradley H.; Hammett, Wilson H.; Jackson, Courtney; Atkins, Dana L.; Belus, Jennifer M.; Kemp, Christopher G. | Implementation outcomes and strategies for depression interventions in low- and middle-income countries: a systematic review | Global Mental Health |
| 2020 | Werfalli, Mahmoud; Raubenheimer, Peter J.; Engel, Mark; Musekiwa, Alfred; Bobrow, Kirsten; Peer, Nasheeta; Hoegfeldt, Cecilia; Kalula, Sebastiana; Kengne, Andre Pascal; Levitt, Naomi S. | The effectiveness of peer and community health worker-led self-management support programs for improving diabetes health-related outcomes in adults in low- and-middle-income countries: a systematic review | Systematic Reviews |
| 2020 | White, M. C.; Peven, K.; Clancy, O.; Okonkwo, I.; Bakolis, I.; Russ, S.; Leather, A. J. M.; Sevdalis, N. | Implementation Strategies and the Uptake of the World Health Organisation Surgical Safety Checklist in Low and Middle Income Countries: A Systematic Review and Meta-analysis | Ann Surg |
| 2020 | Winchester, D. E.; Merritt, J.; Waheed, N.; Norton, H.; Manja, V.; Shah, N. R.; Helfrich, C. D. | Implementation of Appropriate Use Criteria for Cardiology Tests and Procedures: a Systematic Review and Meta-analysis | Eur Heart J Qual Care Clin Outcomes |
| 2020 | Wolfenden, Luke; Barnes, Courtney; Jones, Jannah; Finch, Meghan; Wyse, Rebecca J.; Kingsland, Melanie; Tzelepis, Flora; Grady, Alice; Hodder, Rebecca K.; Booth, Debbie; Yoong, Sze Lin | Strategies to improve the implementation of healthy eating, physical activity and obesity prevention policies, practices or programmes within childcare services | Cochrane Database Of Systematic Reviews |
| 2019 | Wolfenden, Luke; Reilly, Kathryn; Kingsland, Melanie; Grady, Alice; Williams, Christopher M.; Nathan, Nicole; Sutherland, Rachel; Wiggers, John; Jones, Jannah; Hodder, Rebecca; Finch, Meghan; McFadyen, Tameka; Bauman, Adrian; Rissel, Chris; Milat, Andrew; Swindle, Taren; Yoong, Sze Lin | Identifying opportunities to develop the science of implementation for community-based non-communicable disease prevention: A review of implementation trials | Preventive Medicine |
| 2019 | Wu, Julie Hui‐Chih; Langford, Bradley J.; Daneman, Nick; Friedrich, Jan O.; Garber, Gary | Antimicrobial Stewardship Programs in Long‐Term Care Settings: A Meta‐Analysis and Systematic Review | Journal Of The American Geriatrics Society |
| 2022 | X. L. Liu; T. Wang; J. Y. Tan; S. Stewart; R. J. Chan; S. Eliseeva; M. J. Polotan; I. Zhao | Sustainability of healthcare professionals' adherence to clinical practice guidelines in primary care | Bmc Prim Care |
| 2020 | Yoong, Sze Lin; Hall, Alix; Stacey, Fiona; Grady, Alice; Sutherland, Rachel; Wyse, Rebecca; Anderson, Amy; Nathan, Nicole; Wolfenden, Luke | Nudge strategies to improve healthcare providers' implementation of evidence-based guidelines, policies and practices: a systematic review of trials included within Cochrane systematic reviews | Implementation Science |
