# Supplementary material for: A systematic review of experimentally tested implementation strategies across health and human service settings: evidence from 2010-2022
Source: Implement Sci. 2024 Jun 24;19:43. doi: 10.1186/s13012-024-01369-5 (PMC11194895; doi:10.1186/s13012-024-01369-5)
Supplement: Supplementary file 4 — Supplementary Material 4. [file 13012_2024_1369_MOESM4_ESM.docx]

| **Additional File 4. ERIC- Ashcraft et al., Implementation Strategies (2023)** | | |
| --- | --- | --- |
| **#** | **ERIC Implementation Strategies** | **2023 Implementation Strategies** |
| Use evaluative and iterative strategies | | |
| 4 | Assess for readiness and identify barriers and facilitators | Same |
| 5 | Audit and provide feedback | Same |
| 14 | Conduct cyclical small tests of change | Same |
| 18 | Conduct local need assessment | Same |
| 23 | Develop a formal implementation blueprint | Same |
| 26 | Develop and implement tools for quality monitoring | Same |
| 27 | Develop and organize quality monitoring systems | *Included in Develop Implementation tools for Quality Monitoring* |
| 46 | Obtain and use patients/consumers and family feedback | Same |
| 56 | Purposefully reexamine the implementation | Same |
| 61 | Stage implementation scale up | Same |
| New |  | Assess and redesign workflows |
| Provide interactive assistance | | |
| 8 | Centralize technical assistance | Same |
| 33 | Provide Implementation Facilitation | *Broken into Internal Facilitation and External Facilitation* |
| 53 | Provide clinical supervision | Same |
| 54 | Provide local technical assistance | Same |
| New |  | Internal Facilitation |
| New |  | External Facilitation |
| New |  | Create an online learning community |
| Adapt and tailor to context | | |
| 51 | Promote adaptability | Same |
| 63 | Tailor strategies | Same |
| 67 | Use data experts | Same |
| 68 | Use data warehousing techniques | Same |
| Develop stakeholder interrelationships | | |
| 6 | Build a coalition | Same |
| 7 | Capture and share local knowledge | Same |
| 17 | Conduct local consensus discussions | Same |
| 24 | Develop academic partnerships | Same |
| 25 | Develop an implementation glossary | *Included in Distribute Educational Materials* |
| 35 | Identify and prepare champions | Same |
| 36 | Identify early adopters | Same |
| 38 | Inform local opinion leaders | Same |
| 40 | Involve executive boards | Same |
| 45 | Model and simulate change | Same |
| 47 | Obtain formal commitments | Same |
| 48 | Organize clinician implementation team meetings | Same |
| 52 | Promote network weaving | Same |
| 57 | Recruit, designate, and train for leadership | Same |
| 64 | Use advisory boards and workgroups | Same |
| 65 | Use an implementation advisor | *Included in Implementation Facilitation (Internal & External)* |
| 72 | Visit other sites | Same |
| New |  | Engage community resources outside the practice |
| Train and educate stakeholders | | |
| 15 | Conduct educational meetings | Same |
| 16 | Conduct educational outreach visits | Same |
| 19 | Conduct ongoing training | Same |
| 20 | Create a learning collaborative | Same |
| 29 | Develop educational materials | *Included in either Conduct Educational Meetings or Distribute Educational Materials* |
| 31 | Distribute educational materials | Same |
| 43 | Make training dynamic | Same |
| 55 | Provide ongoing consultation | Same |
| 60 | Shadow other experts | Same |
| 71 | Use train-the-trainer strategies | Same |
| 73 | Work with educational institutions | *Included in Develop Academic Partnerships* |
| Support clinicians | | |
| 21 | Create new clinical teams | Same |
| 30 | Develop resource sharing agreements | Same |
| 32 | Facilitate relay of clinical data to providers | Same |
| 58 | Remind clinicians | Same |
| 59 | Revise professional roles | Same |
| Engage consumers | | |
| 37 | Increase demand | Same |
| 39 | Intervene with patients/consumers to enhance uptake and adherence | Same |
| 41 | Involve patients/consumers and family members | Same |
| 50 | Prepare patients/consumers to be active participants | Same |
| 69 | Use mass media | Same |
| Utilize financial strategies | | |
| 1 | Access new funding | Same |
| 2 | Alter incentive/allowance structures | Same |
| 3 | Alter patient/consumer fees | Same |
| 28 | Develop disincentives | Same |
| 34 | Fund and contract for the clinical innovation | Same |
| 42 | Make billing easier | Same |
| 49 | Place innovation on fee for service lists/formularies | Same |
| 66 | Use capitated payments | Same |
| 70 | Use other payment schemes | Same |
| Change infrastructure | | |
| 9 | Change accreditation or membership requirements | Same |
| 10 | Change liability laws | Same |
| 11 | Change physical structure and equipment | *Included in Change Record Systems* |
| 12 | Change record systems | Same |
| 13 | Change service sites | Same |
| 22 | Create or change credentialing and/or licensure standards | *Included in Change Record Systems* |
| 44 | Mandate change | Same |
| 62 | Start a dissemination organization | Same |
